# Reprogramming the tumor microenvironment in mesothelioma: from immune suppression to therapeutic opportunity

**DOI:** 10.3389/fimmu.2026.1814514

**Published:** 2026-10-06

**Authors:** Luigi Cerbone, Alessia Garau, Andrea Balossino, Simona Martinotti, Elia Ranzato, Carlo Genova, Valentina Rigo, Stefano Meda, Silvia Ravera, Hector Josè Soto Parra, Maria Carmela Piccirillo, Alessandra Gurrera, Alberto Terminella, Mattia Manitto, Michela Croce, Simona Coco, Federica Grosso

**Affiliations:** 1 SSD Mesotelioma, Melanoma e Tumori Rari, IRCCS AOU SS Antonio e Biagio e Cesare Arrigo, Alessandria, Italy; 2 DISSTE - Dipartimento per lo Sviluppo Sostenibile e la Transizione Ecologica, Università del Piemonte Orientale, Vercelli, Italy; 3 SC Infrastruttura Ricerca Formazione e Innovazione, Dipartimento Attività Integrate Ricerca e Innovazione (DAIRI), IRCCS AOU SS Antonio e Biagio e Cesare Arrigo, Alessandria, Italy; 4 UO Tumori Polmonari, Oncologia Medica, AOM - IRCCS Ospedale Policlinico San Martino, Genova, Italy; 5 DiSIT- Dipartimento di Scienze e Innovazione Tecnologica, Università del Piemonte Orientale, Alessandria, Italy; 6 SSD Laboratori di Ricerca, Dipartimento Attività Integrate Ricerca e Innovazione (DAIRI), IRCCS AOU SS Antonio e Biagio e Cesare Arrigo, Alessandria, Italy; 7 UOC Clinica di Oncologia Medica, AOM - IRCCS Ospedale Policlinico San Martino, Genova, Italy; 8 DIMI - Dipartimento di Medicina Interna e Specialità Mediche, Università degli Studi di Genova, Genova, Italy; 9 UOC Bioterapie, AOM - IRCCS Ospedale Policlinico San Martino, Genova, Italy; 10 SC Chirurgia Toracica, IRCCS AOU SS Antonio e Biagio e Cesare Arrigo, Alessandria, Italy; 11 SC Malattie Apparato Respiratorio, IRCCS AOU SS Antonio e Biagio e Cesare Arrigo, Alessandria, Italy; 12 UO Oncologia Medica, Azienda Ospedaliero Universitaria Policlinico “G. Rodolico-S. Marco”, Catania, Italy; 13 SC Sperimentazioni Cliniche, Istituto Nazionale Tumori IRCCS - Fondazione G. Pascale, Napoli, Italy; 14 UOC Anatomia Patologica, Azienda Ospedaliero Universitaria Policlinico “G. Rodolico-S.Marco”, Catania, Italy; 15 UOC Chirurgia Toracica, Azienda Ospedaliero Universitaria Policlinico “G.Rodolico-S. Marco”, Catania, Italy; 16 SC Chirurgia Toracica, AOM - IRCCS Ospedale Policlinico San Martino, Genova, Italy

**Keywords:** immune checkpoint inhibitors, immune-signatures, macrophage, pleural mesothelioma, tumor microenvironment

## Abstract

Pleural mesothelioma (PM) is a rare and aggressive cancer with limited treatment options and poor prognosis. Unlike other thoracic cancers responding well to immunotherapy, PM typically lacks key predictive biomarkers such as Programmed Death-Ligand 1 (PD-L1) tumor expression. Nonetheless, immune checkpoint inhibitors (ICIs) have shown clinical activity in PM, particularly in non-epithelioid subtypes. However, only a subset of patients benefits from this approach, and resistance remains a major challenge. A growing body of evidence suggests that tumor microenvironment (TME) plays a central role in shaping this resistance. In PM, the TME is notably immunosuppressive, dominated by regulatory T cells, myeloid-derived suppressor cells, and tumor-associated macrophages. These elements act as a shield against immune activation, limiting the efficacy of ICIs. Novel therapeutic strategies aimed at depleting or modulating suppressive immune populations within the TME are emerging as promising avenues to overcome resistance and enhance the effectiveness of ICIs. In this narrative review, we examine the unique features of the TME in PM, its role in immunotherapy resistance, and emerging strategies aimed at harnessing or remodeling the TME to improve patient outcomes.

## 1 Introduction

Pleural mesothelioma (PM) is a rare cancer arising from the epithelial lining enveloping the lungs, associated with workplace or environmental asbestos exposure with a dismal prognosis (1). Indeed, most patients die within two years from diagnosis (2). The therapeutic landscape of PM has dramatically changed in the past few years. First, the role of radical surgery has been substantially redefined. Indeed, MARS2, a randomized clinical trial of extended pleurectomy decortication versus first line chemotherapy, reported worse overall survival in the surgery arm (3), albeit its results remain debated (4, 5). Second, immune checkpoint inhibitors (ICIs) have reshaped the treatment scenario of PM (2). Indeed, recently, two clinical trials exploring ICI combination strategies have modified clinical practice in PM. First of all, the Checkmate 743 trial (6) demonstrated that the combination of ipilimumab and nivolumab prolonged overall survival (OS) versus platinum- pemetrexed chemotherapy in first line unresectable PM. Furthermore, the IND 227 trial (7) proved that the addition of pembrolizumab to standard platinum pemetrexed chemotherapy significantly prolonged OS in patients with unresectable PM. In both trials, patients with non-epithelioid histology derived the greatest clinical benefit from ICI-based strategy. However, most PM patients do not benefit from ICIs.

PM has generally a low tumor mutational burden (TMB). In addition, the tumor immune microenvironment of PM is highly immunosuppressive as it is enriched with immunosuppressive M2 macrophages, regulatory T cells (Treg) and cells expressing Programmed Death-Ligand 1 (PD-L1) (8). In addition, acquired resistance to ICIs remains one of the most relevant challenges. Currently, a growing body of evidence suggests that the tumor microenvironment (TME) plays a central role in shaping treatment resistance. Recent advances suggest that TME is not merely a barrier but also a modifiable component of the tumor ecosystem. In this narrative review, we examine the unique features of the TME in mesothelioma, its role in immunotherapy resistance, and emerging strategies alongside relevant biomarkers such as PD-L1 expression, TMB, and immune gene signatures aimed at harnessing or remodeling the TME to improve patient outcomes. By targeting suppressive immune cell populations and reprogramming the TME, it may be possible to restore effective anti-tumor immunity and extend the benefits of immunotherapy to a broader patient population.

### 1.1 ICIs in PM, biological rationale of differential strategies

As previously mentioned, the introduction of ICIs has reshaped the therapeutic scenario in PM. In particular, the Checkmate 743 and the IND 227/Keynote 483 trials demonstrated that the combination of an anti-CTLA-4 and an anti-PD-1 (Checkmate 743) or the addition of an anti-PD-1 to standard platinum pemetrexed based chemotherapy (IND 227-Keynote 483) improved OS when compared to standard platinum pemetrexed chemotherapy in PM. Despite the ultimate effects of the treatment are similar, both resulting in an increase in the cytotoxic cell effects in PM tumor, with different mechanisms of action. Indeed, ipilimumab acts through the inhibition of CTLA-4, an inhibitory immune checkpoint (ICI) dampening the effects of CD86, a stimulatory molecule involved in naive T cells priming and activation (8). Conversely, chemotherapy alone increases the infiltration of CD8+ and PD-L1+ cells in PM, while the addiction of immunotherapy to the chemotherapy induces an increased infiltration of CD4+ and CD8+ cells at the tumor level (9–12). While five years results of Checkmate 743 demonstrate a survival benefit in all PM patients, regardless of histology (13), the non-epithelioid histology is still likely to derive the most survival benefit from ICIs. TME features of non-epithelioid histology, which is enriched with CD8+ cells, and overexpresses PD-L1, might be the foundation for increased ICI efficacy in this patient subgroup (**Table 1**) (8).

**Table 1:** Comparison of anti-CTLA-4 + anti-PD-1 *vs* anti-PD-1 plus chemotherapy in PM.

Therapeutic approach	Shared mechanism of action	Pivotal trials	Differential mechanism of action	Shared clinical outcome
Dual Checkpoint Blockade *(Anti-CTLA-4 + Anti-PD-1)*	Anti-PD-1: Blockade of the immunomodulatory signal mediated by PD-1 on T cells	Checkmate 743 (6,8,13)	CTLA-4 Inhibition (Ipilimumab): Blocks CTLA-4 to restore the activity of CD86, a key costimulatory molecule required for naive T-cell priming and activation.	Improved OS compared to standard chemotherapy, with the non-epithelioid subgroup (enriched with CD8+ cells and overexpressing PD-L1) deriving the most pronounced benefit.
Chemo-Immunotherapy *(Anti-PD-1 + Chemotherapy)*		IND 227/Keynote 483 (7)	Chemotherapy: immunogenic changes, decrease in MDSC infiltration increases neoantigen release while the addition of anti-PD-1 unleashes the cytotoxic potential of T cells directly at the tumor site.	

## 2 Role of TME in PM prognosis and therapeutic resistance

### 2.1 Background

The TME of PM is characterized by chronic asbestos-driven inflammation, abundant pro-inflammatory mediators, and a hypoxic milieu that promotes tumor aggressiveness and profoundly shapes immune cell phenotype and function (14) (**Figure 1**). In particular, PM is initiated by asbestos exposure, through immune-mediated mechanisms: mesothelial cells exposed to asbestos fibers secrete inflammatory cytokines that recruit macrophages (15, 16). Inefficient fiber clearance by activated phagocytic macrophages triggers the release of persistent pro-inflammatory cytokines (*i.e.*, TNFα, IL-1β), which drives mesothelial cell transformation (17, 18). Transformed mesothelial cells then remodel the microenvironment by recruiting and polarizing tumor-associated macrophages (TAMs) toward an M2-like CD163^+^ phenotype and promoting the accumulation of myeloid-derived suppressor cells (MDSCs) along with the Tregs, establishing immunosuppression (19, 20) and T cell exhaustion, finally resulting in a reduction in cytotoxic T lymphocytes (CTLs). Given the abundance of M2-macrophages (TAM-M2) and MDSC (21), and a reduced infiltration of T and NK lymphocytes, together with neutrophils, and fibroblasts (22), the TME of PM is generally deemed as “cold”. More specifically, the few CTLs are either present but functionally anergic, reflecting an “immunosuppressed phenotype”, or they are confined to the tumor margins and unable to infiltrate the tumor core, consistent with an “immune-excluded phenotype” (23).

**Figure 1:**
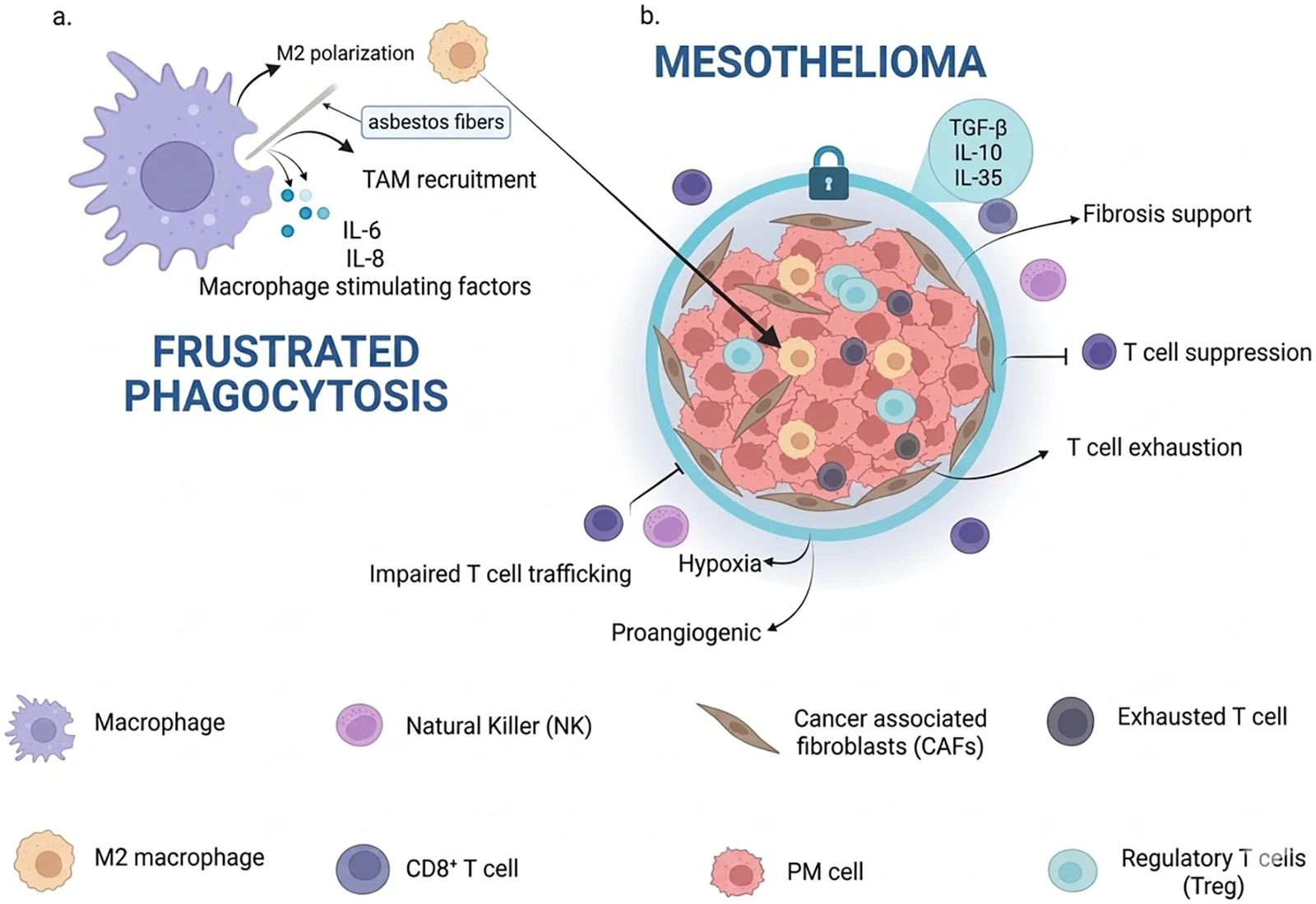
PM microenvironment. **(a)** Asbestos fibers trigger a chronic inflammatory response because macrophages cannot scavenge them, leading to a persistent release of pro-inflammatory cytokines. **(b)** PM TME is dominated by suppressive cells that exclude or exhaust cytotoxic T cells.

Interestingly, the TME differs markedly between two histotypes such as epithelioid and non-epithelioid PM, and these differences reflect patients’ prognosis and response to immunotherapy. Comparatively, epithelioid PM is characterized by a more immunogenic TME, with higher proportions of plasmacytoid dendritic cells (DCs), CD20^+^ B cells, CD4^+^ helper T cells, and exhausted CD8^+^ tumor-infiltrating lymphocytes (TILs), but spatially compartmentalized, with TILs accumulated in the stroma rather than tumor nests defined as “immune-excluded phenotype” (8, 24) (**Table 2**, **Figure 2**). Contrarily, the sarcomatoid PM TME displays abundant TILs but is dominated by suppressive myeloid and Treg populations and impaired antigen processing/presentation and cytotoxic immune activity, defined as “immune-infiltrated yet suppressive” phenotype (8, 24), whereas the Biphasic PM shows a hybrid and heterogeneous TME due to the coexistence of both epithelioid-like and sarcomatoid-like regions.

**Table 2:** TME characteristics and differences across PM histological subtypes.

Subtype	Immune infiltration	Dominant cells	Functional state
Epithelioid	Moderate-high	T cells, B cells, DCs	Immune-active but partly excluded
Sarcomatoid	High	TAMs, Tregs, neutrophils	Immune-suppressive/pro-tumor
Biphasic	Variable	Mixed	Spatially heterogeneous

**Figure 2:**
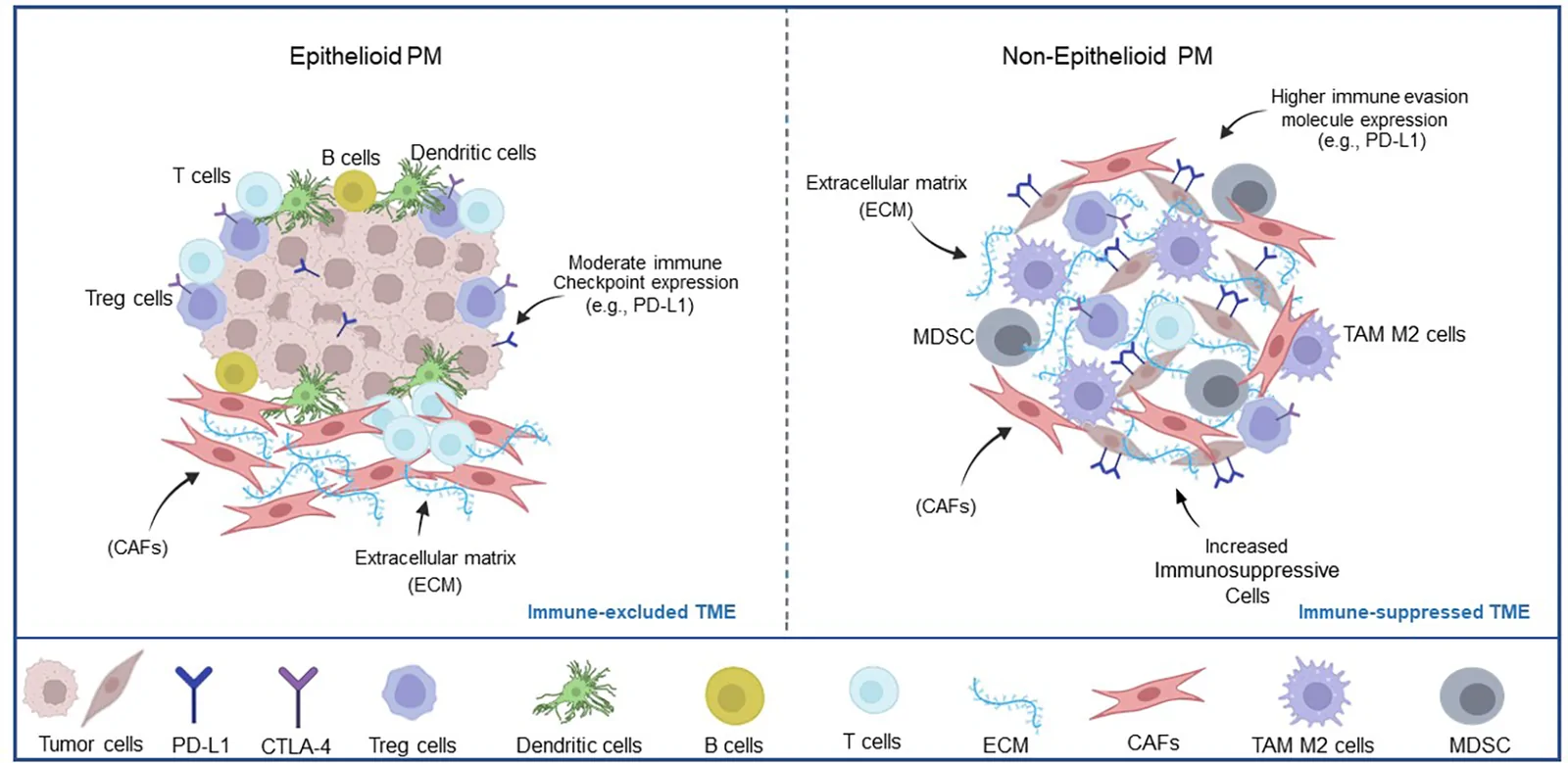
Graphical representation of the TME in PM. Epithelioid PM is characterized by an immune-excluded TME, whereas non-epithelioid PM displays a highly immunosuppressive TME, with tumor cells expressing higher levels of PD-L1. CAFs, cancer-associated fibroblasts; ECM, extracellular matrix, Treg: regulatory T cells; MDSC, myeloid-derived suppressor cells; TAM M2, tumor-associated macrophages M2 subset.

Currently, one of the major challenges in dissecting the TME in PM is high intratumoral heterogeneity, and substantial efforts have been made to identify alternative and more accessible sources for TME analysis, among which pleural effusion (PE) represents a particularly relevant option. Indeed, the pleural fluid derived from routinely performed palliative pleural evacuations provides a unique repository of TME. However, the composition and functional state of immune cells within the tumor may differ significantly from those found in PE. Indeed, in a study conducted comparing biopsy with PE from the same PM patients, high intratumor Tregs, together with high MDSC, were associated with worse PFS and OS. In contrast, immune cell populations characterized in matched PE fluid lacked significant prognostic correlation (25).

### 2.2 Immune cell exhaustion

T-cell exhaustion is a dysfunctional state arising from prolonged or chronic antigen exposure, characterized by the upregulation of inhibitory receptors (immune checkpoints, ICs) that deliver suppressive signals (**Figure 3**). This process leads to reduced T-cell activation, diminished cytotoxicity, and impaired effector functions, but simultaneously provides opportunities for targeted immunotherapeutic interventions.

**Figure 3:**
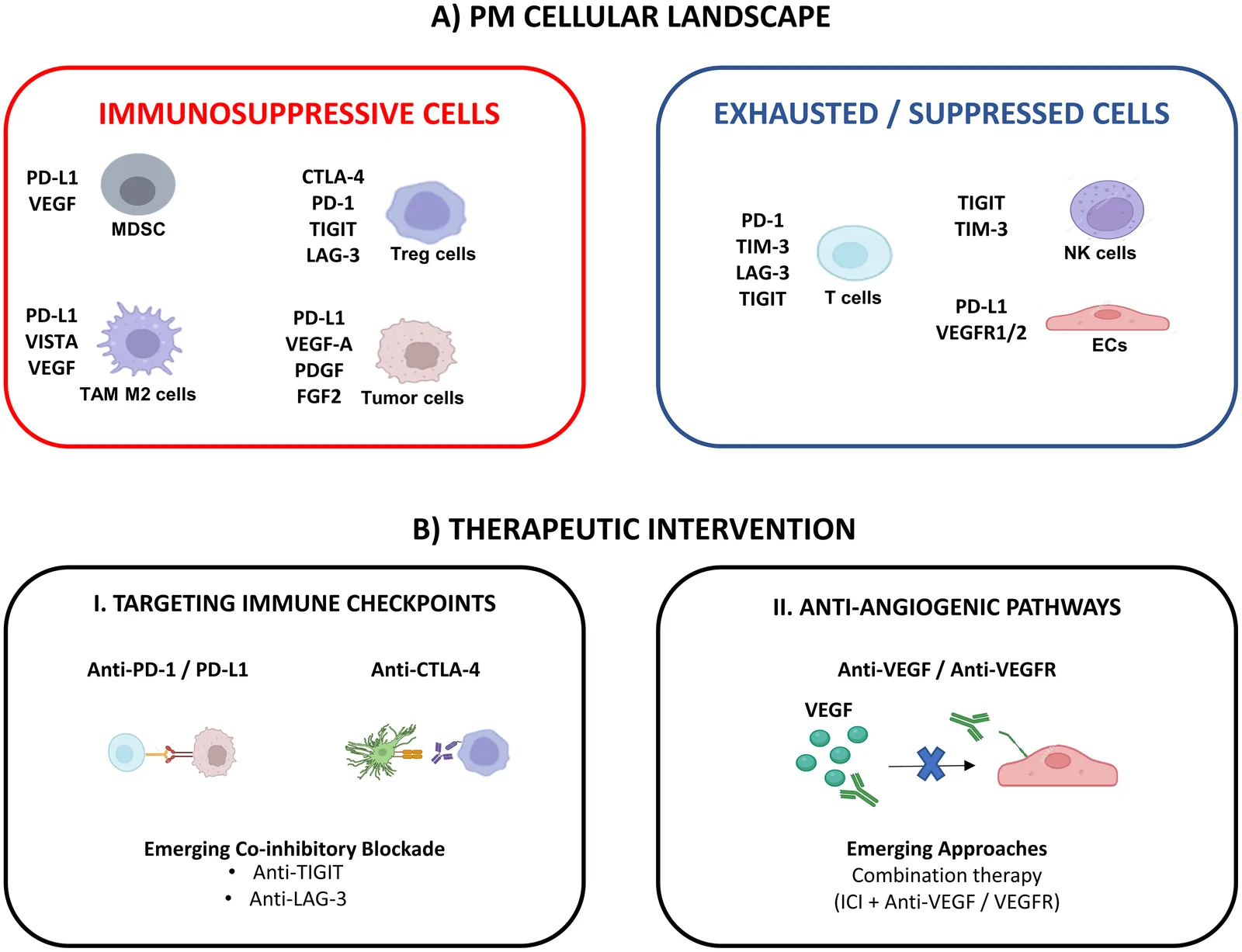
The PM TME and TME-driven therapeutic strategies. The **(A)** illustrates the complex cellular landscape of PM, characterized by immunosuppressive regulators (Tregs, MDSCs, M2 macrophages) and exhausted effector cells (CD8+ T cells, NK cells) expressing multiple immune checkpoints (PD-1, VISTA, CTLA-4, TIGIT, LAG-3, TIM-3). The presence of endothelial cells (ECs) and pro-angiogenic factors (VEGF, PDGF, FGF2) promotes physical and functional immune exclusion. The **(B)** summarizes current therapeutic interventions: (I) Targeting immune checkpoints (ICIs) to restore T-cell activity; (II) Reprogramming the myeloid compartment to alleviate suppression; and (III) Inhibiting angiogenic pathways to enhance immune penetration and overcome therapy resistance.

Anergic T cells express IC receptors, resulting in ineffective antitumor activity and favoring tumor growth (26). Multiplexed imaging mass cytometry performed on tumor tissues from 15 patients pre- and post-(anti-PD-1 + anti-CTLA-4) ICIs treatment (NCT02592551) revealed that the proximity of CD4+, CD8+, and DCs were associated with clinical responsiveness (27). CD8+ T cells expressed CD45RO, PD-1, and high levels of IFNγ and granzyme B. Among CD4+ T cells, the authors observed a significantly lower presence of regulatory FOXP3+CD4+ Tregs. Notably, it is known that the presence, in TME, of CD8+ T cells is a prerequisite for ICI efficacy; indeed, high CD8+ T cell density and their proximity to tumor cells were associated with better response of PM patients to nivolumab (*i.e*., anti-PD-1) (28). A recent study in a small cohort of PM patients (19/22 with epithelioid histology), treated with different chemotherapy regimens, reported a correlation between higher densities of macrophages and CD4^+^ T lymphocytes and longer survival (29). Unexpectedly, no significant association was found for CD8^+^ T cells, despite their frequencies being comparable to those of CD4^+^ cells, and the expression of high levels of Granzyme B.

PM tumors also exploit IC pathways to escape immune surveillance; particularly, *BAP1*-deficient PM tumors exhibited increased expression of PD-L1, PD-1, and Lymphocyte Activation Gene 3 (LAG-3) (30).

PD-L1 expression has been observed on both tumor and immune infiltrate cells, including TAMs and DCs, and its expression has been associated with a poorer prognosis (29, 31). It has been proposed that chemotherapy treatment might enhance PD-L1 expression on PM cells. Data from the KEYNOTE-02 and the JAVELIN trial in PM indicate that PD-L1 expression can be seen irrespective of prior chemotherapy treatment (32, 33); indeed, in the JAVELIN trial, both PD-L1 positive and negative (<5% of PD-L1 expressing cells) patients responded to an anti-PD-L1 (i.e., Avelumab) therapy with durable antitumor activity (33). Marcq and colleagues using a cut-off ≥ 1% of positive tumor cells found that 68% of chemotherapy-untreated patients showed positivity to PD-L1 expression (34). Sarcomatoid histology also shows a higher degree of positivity for PD-L1 than the epithelioid and biphasic subtypes (34), even if a recent study on 203 PM patients did not find an association between PD-L1 expression (>1%) of tumor cells and histological subtype (35). However, in the same study, >10% of tumor cells expressing PD-L1 predicted worse OS in PM. Nevertheless, variability in PD-L1 tumor positivity across studies makes it challenging to determine whether PD-L1 serves as a prognostic or predictive biomarker in PM (16, 34). The presence of PD-L1+ tumor cells and both PD-1+ and PD-L1+ infiltrating immune cells has also been described in the PE of patients (36).

In addition, soluble (s) forms of PD-L1 are detected in PE of PM. In one study (n = 77), higher sPD-L1 levels were associated with worse OS (37), whereas in another study (n = 84), no significant association was observed (38). Also, in a retrospective analysis on 171 patients with advanced solid tumors, comprising 6 PM, receiving anti-PD-1 (*i.e.*, nivolumab or pembrolizumab), high circulating levels of sPD-L1 in combination with low levels of sPD-1 were defined as a potential negative biomarker of therapy efficacy and associated with worse PFS and OS (39). In the NIBIT-MESO-1 study involving 40 patients with PM receiving anti-PD-L1 (*i.e.*, durvalumab) in combination with anti-CTLA-4 (*i.e.*, tremelimumab), circulating sPD-L1 levels increased significantly during therapy. Patients with baseline sPD-L1 levels below a predefined cut-off showed a longer OS; moreover, a higher fold-change in sPD-L1 during treatment was also associated with improved OS (40).

PD-1 is expressed on tumor cells (34) as well as on tumor-infiltrating lymphocytes, especially CD8^+^, CD4^+^ T cells, and some Tregs within the TME. PD-1 expression on PM-infiltrating lymphocytes was detected by immunohistochemistry in 50% of a total of 164 patients, and high PD-1 expression (on more than 10% of tumor-infiltrating lymphocytes) was seen only in 24% (39 patients). PD-1 >1%, or >10% did not prove to be prognostic in terms of OS nor related to histological subtype (35). Patients with tumors simultaneously harboring PD-1+T cells and PD-1+macrophages benefited the most from anti-PD-1 (*i.e*., pembrolizumab) immune therapy (41). In a study of 58 patients with PM who underwent extrapleural pneumonectomy, PD-1 expression on TILs was high in 41/58 cases (~70.7%), as determined by IHC. Paradoxically, the loss of HLA class I was associated with a better prognosis (HR = 0.35; p-value=0.011). Additionally, the combination of CD8 negativity with high HLA class I expression (CD8-neg/HLA-high) was strongly associated with worse prognosis in stage I–II cases (HR ~4.635; p-value = 0.0006) (42). PD-1 expression was also found on both CD4+ and CD8+ T cells from PE and their proportions were significantly higher than in peripheral blood (43).

In BRCA1-associated protein 1 (*BAP1)*-deficient PM, the presence in the TME of TCF1+/PD-1+/CD8+ precursor-exhausted T cells is associated with increased CD163+ M2 macrophages and reduced efficacy of ICIs (30), implying the instauration of an immune suppressive/exhausted TME. This context suggests a possible reduction in the efficacy of ICI therapy, although this has not yet been demonstrated in clinical trials.

CD70, a member of the TNF superfamily, is expressed on B, T, and DCs, and interacts with CD27, providing an important co-stimulus for T cell activation and proliferation (44). Since it is aberrantly overexpressed in diverse malignancies, including PM, it is being considered a target for diverse therapies (*i.e.*, CAR‐T, CAR‐NK, monoclonal antibodies, and antibody‐drug conjugates) (45). The OS of PM patients was negatively affected by those PM co-expressing CD70 and PD-L1 (14 double positive in a cohort of 171 patients comprising all histologies) (46).

While PD-1 is a well-known immune checkpoint receptor, TIM-3 (T-cell immunoglobulin and mucin-domain containing-3) is emerging as a potential therapeutic target; it is expressed on various immune cells, characterizing exhausted CD8^+^ T cells, Tregs, NK cells, and some myeloid cells (47). In PM, particularly in the sarcomatoid subsets, infiltrating T cells display PD-1 and TIM-3 expression, highlighting the possibility of specific ICI targeting as a new immunotherapeutic approach (48).

CTLA-4 which is primarily expressed on Tregs, inhibits co-stimulatory signaling, fundamental for effective T-cell activation (49). Targeting CTLA-4 with high doses of tremelimumab in patients resistant to chemotherapy with advanced PM showed that some patients had immune-related objective responses (50), sustaining the role of CTLA-4 inhibition, and supporting the use of a combination of anti-PD-1 and anti-CTLA-4 ICIs. Subsequent studies *(i,e.*, the combination of tremelimumab + durvalumab) have further explored CTLA-4 + PD-L1/PD-1 blockade in PM (51). A translational analysis from CheckMate-743 found that combination CTLA-4 + PD-1 therapy strongly induces proliferation and activation of memory CD4 and CD8 T cells, but it did not find a clear depletion of Tregs (52). The same trial showed greater survival benefit in non-epithelioid (sarcomatoid/biphasic) PM compared with chemotherapy (53). Indeed, evidence suggests differences in CTLA-4 expression between epithelioid and non-epithelioid (sarcomatoid) PM. For instance, a study of 45 PM cases reported higher CTLA-4 immunohistochemical expression in the epithelioid subtype compared to sarcomatoid (54). However, the available literature remains limited, and further investigation is needed to confirm and clarify these subtype-specific differences.

TIGIT (T cell immunoreceptor with Ig and ITIM domains) is an IC receptor expressed mainly on activated T cells, Tregs, and NK cells (55). In PM, tumor-infiltrating lymphocytes show elevated expression of TIGIT, including on both Tregs and CD8+ T cells; high numbers of Tregs expressing TIGIT in PM are associated with tumor-infiltrating lymphocytes hypo-functionality (56).

CD276 (B7-H3), a member of the B7 family, is an IC ligand and is considered a potential therapeutic target due to its expression on tumor cells. Under normal conditions, CD276 is involved in preventing autoimmunity. In cancer, it is expressed on both cancer cells and some activated tumor-infiltrating immune cells, which exerts inhibitory effects on cytotoxic T and NK cells (57). A recent study using expression profiles of 86 PMs from TCGA database revealed that CD276 mRNA was highly expressed in PM compared to normal tissue, and was associated with poor prognosis in epithelioid PM, being an independent prognostic factor for this subset of patients (58).

Lymphocyte-activation gene 3 (LAG-3) is another IC molecule implicated in immune regulation within the TME. In PM significant positive correlation between tumor-infiltrating NK cells and LAG-3 expression levels has been demonstrated by multiplex immune fluorescence on tissue microarray slices (59). In addition, elevated *LAG3* mRNA (TCGA, n=86) and protein (IHC, n=38) expression were correlated with a better prognosis in PM (60), with the epithelioid subset showing the highest *LAG3* gene expression in TCGA. However, the role of LAG-3 on PM prognosis or survival seems contradictory. In a study of 275 patients with an initial clinical diagnosis of PM, the expression of PD-1, LAG-3, and TIM-3 in TILs both in tissue and PE from the same patient correlated with lower OS. In particular, only the tumor-infiltrating CD4+ subset expressing PD-1, LAG-3, and TIM-3 was considered a negative prognostic factor (25). Marcq et al. showed an abundance of T cells expressing TIM-3, LAG-3, and PD-1 in PM from PM patients; also, NK cells showed positivity for the abovementioned IC, mainly TIM-3 and LAG-3, giving insights for developing effective immunotherapy (61).

V-domain Ig suppressor of T-cell activation (VISTA) is a member of the B7 family and is expressed primarily on myeloid cells and T-lymphocytes; it acts negatively on T cell activation, proliferation, and cytokine production and is considered in the TME quite a potential mechanism of tumor immune evasion (62, 63). In PM, VISTA expression was detected on a publicly available dataset, TCGA (64), and then confirmed by IHC (65). In the latter study, a higher VISTA expression was associated with better OS (65). In addition, higher gene expression levels of VISTA together with LAG-3 derived from TCGA database (30, 66), were associated with a PD-1 therapy response signature in PM tumors carrying alterations in the *BAP1* only. Given the high expression reported and the association with better outcome, VISTA may still represent a promising IC target in epithelioid mesothelioma.

Finally, the CD94/NK group 2 member A (NKG2A) a further co-inhibitory immune checkpoint has been found expressed on both NK cells and T cells that binds to a nonclassical minimally polymorphic HLA class I molecule (HLA-E), and transduces inhibitory signaling, suppressing NK and T cytokine secretion and cytotoxicity (67). PM expresses HLA-E and NKG2A/HLA-E interaction between NK/T and tumor cells may potentially become an important new therapeutic axis in PM, especially for epithelioid histology (68).

### 2.3 Tregs

In PM specimens, CD4^+^ T cells that express the FOXP3 transcription factor, characterizing the Tregs subset, are frequently found. The proportion of CD4^+^FOXP3^+^ cells varies broadly (*i.e.*, from ~1% to 50% in one study) suggesting a potential immunosuppressive component in the TME (21, 34). Tregs are natural occurring cells essential for maintaining immune tolerance and preventing autoimmunity by suppressing immune responses via different mechanisms, such as: *i)* inhibitory cytokines (i.e., TGF-β, IL-10, and IL-35); *ii)* sequestering T cell growth factor IL-2 via high expression of CD25, depriving effector cells of a key growth factor; *iii)* converting ATP to immunosuppressive adenosine via CD39 and CD73 molecules; and *iv)* downregulating co-stimulatory molecules (CD80/CD86) on DCs via CTLA-4 (56). In cancer, Tregs accumulate in the TME, where, induced by cancer cells, they exert their functions, resulting in suppression of the antitumor immunity by inhibiting CTL activation, thereby facilitating immune evasion and tumor progression (69). In PM, Tregs have been found in tumors as well as in PEs, and the ratio of CD4^+^ T cells to Tregs in tumor tissues was associated with OS; in particular, a higher CD4^+^/Treg ratio was significantly associated with longer OS (particularly for epithelioid compared to non-epithelioid (70), whereas the non-epithelioid tumors had distinct Treg-related patterns (19, 21, 70). Some studies suggest that Treg infiltration is associated with poorer survival in non-epithelioid (sarcomatoid/biphasic) PM (16, 70), but, to date, no published study has definitively established differences in Treg densities between epithelioid and non-epithelioid PM.

The co-presence of high intratumor Tregs, together with high MDSC, has been associated with worse PFS and OS in PM (25). Marcq and colleagues identified the expression of FOXP3 CD4+ TILs in the stroma of 80% of formalin-fixed paraffin-embedded (FFPE) tumor samples from untreated and 56% of cisplatin and/or pemetrexed pretreated PM patients, suggesting a negative effect of chemotherapy drugs on Treg cells population, probably related to the specific chemotherapy drug (34). Indeed, low-dose cyclophosphamide given to PM patients together with an immunotherapeutic vaccine based on DCs led to a significant decrease of Tregs in patients’ peripheral blood (71).

Currently, several preclinical studies have investigated different strategies to target these immunosuppressive cells. In a preclinical syngeneic model of PM, the efficient depletion of FOXP3+ Tregs by diphtheria toxin injection, immediately after nonablative oligofractionated irradiation, resulted in long-term cure (72). Targeting Tregs by CTLA-4 blockade combined with chemotherapy resulted in tumor growth delay in a murine mesothelioma model; the addition of radiation therapy to enhance antitumor activity to anti-CTLA-4 blockade showed synergistic efficacy (73). Moreover, in an orthotopic murine model of PM, the combination of CTLA-4 blockade with chemotherapy (i.e., pemetrexed) exerted a synergistic antitumor effect resulting in prolonged survival through the reshaping of TME from immune suppressive to immune responsive, where CD8+ T cells were the main immune cell population endowed with antitumor activity (74).

Taken together, these observations suggest that effective PM therapy may require strategies that go beyond classical ICIs and explicitly target the immunosuppressive niche dominated by Tregs.

### 2.4 Myeloid-derived suppressor cells

MDSCs are immature immune cells that expand in cancer and act as powerful brakes on the immune system. They actively support tumor growth and spread by releasing enzymes and molecules (*i.e.*, arginase, nitric oxide, prostaglandins, and reactive oxygen species) that starve or disable T cells and by producing factors such as VEGF and MMP9 that promote angiogenesis and prepare distant sites for metastasis (75). In PM, MDSCs are recruited into the tumor, where they block T-cell activity, supporting PM growth (**Figure 3**). They also correlate with diminished T-cell activity and worse overall clinical outcomes (lower PFS and OS) (16, 25, 76). No published study to date clearly demonstrates a difference in MDSC density between epithelioid and non-epithelioid PM. It should be noted that while MDSCs are well documented in tumor tissue, data on their recruitment into the PE compartment and their prognostic impact therein are less robust. Higher frequencies of MDSCs in PE and tissue were detected in PM compared to pleuritis; high intra-tumor levels of MDSCs (together with Tregs) are associated with worse PFS and OS (25). A recent integrative study has shown that PM tumors with low *BAP1* expression had decreased abundance of CD14^+^ MDSCs compared with *BAP1*-high tumors (30). Higher percentages of MDSCs in PE were associated with lower CD8^+^ T cell cytotoxicity and reduced IFN-γ production. This suggests that MDSCs may contribute to the immunosuppressive environment of PEs, although other studies are needed to clarify their mechanism (77). In the NVALT19-trial, in which gemcitabine maintenance was tested versus best supportive care after a standard first-line treatment, patients receiving gemcitabine showed significantly prolonged PFS and lower MDSC and Tregs frequencies and increased T-cell proliferation (78, 79). The Cyclooxygenase-2 (COX-2)/prostaglandin E2 (PGE2) signaling axis has emerged as a key regulator of MDSC expansion and immunosuppressive activity within the TME. In particular, COX-2 is an inducible enzyme that converts arachidonic acid into prostaglandin H_2_, the precursor of PGE2, a potent immunomodulatory molecule (80). In PM, COX-2 is overexpressed, leading to elevated PGE2 levels in the TME, and correlates with poor prognosis (81). PGE2 skews differentiation toward MDSCs, which express COX-2 and produce PGE_2_, reinforcing the immunosuppressive milieu in a positive loop (82). In a syngeneic murine mesothelioma model, COX-2 inhibition using Celecoxib reduced the number and suppressive function of MDSCs, particularly the granulocytic MDSC subset, and improved DC-based immunotherapy (76). Moreover, eliminating granulocytic MDSCs during treatment with a modified vaccinia virus Tiantan enhanced the generation of antitumor cytotoxic T lymphocytes, resulting in the clearance of established mesothelioma lesions and resistance to subsequent tumor rechallenge (83). Finally, preclinical studies support the potential of MDSC-targeted therapies, particularly when used in combination regimens, to improve outcomes in PM.

In addition to preclinical evidence, emerging clinical data support the relevance of MDSCs as predictive biomarkers for ICI efficacy. In the 5-year update of the CheckMate 743 trial evaluating first-line nivolumab plus ipilimumab in unresectable PM, baseline peripheral blood monocytic MDSC (M-MDSC) levels were identified as an independent negative predictive biomarker of clinical outcome; the increase in log-transformed baseline circulating M-MDSC levels in patients treated with nivolumab plus ipilimumab, was associated with a significantly higher risk of death (HR = 1.25, 95% CI 1.09–1.43), with higher M-MDSC levels also correlating with shorter PFS (13). These findings provide important clinical validation of the immunosuppressive role of M-MDSCs and highlight peripheral blood M-MDSC quantification as a promising, minimally invasive biomarker for patient stratification and treatment selection. In addition, these data strengthen the biological rationale for therapeutic strategies targeting the COX-2/PGE2–MDSC axis, as preclinical studies have shown that COX-2/PGE2 inhibition can reduce MDSC accumulation and suppressive activity, thereby potentially enhancing responsiveness to ICI.

### 2.5 Macrophages

TAMs are a predominant and functionally versatile immune population within the TME of PM, influencing tumor progression, immune evasion, and therapeutic response (20, 29). TAMs primarily originate from circulating monocytes, which differentiate into macrophages upon migration to the target tissue (84). In PM, macrophages are among the first immune populations recruited in response to asbestos exposure, the key etiological factor in PM pathogenesis. Indeed, when asbestos fibers reach the pleura, plenty of macrophage attractants, including IL-6, IL-8 and macrophage-stimulating factors, are released, establishing a robust inflammatory response. However, macrophages attempting to scavenge asbestos fibers undergo a process known as “frustrated phagocytosis” as the fibers’ dimensions and mineral composition prevent an effective clearance. This process triggers a chronic inflammatory state characterized by a sustained cytokine cascade, reactive oxygen species production, tissue hypoxia with consequent DNA damage, and gene alterations. All these molecular events lead to fibrosis, collagen deposition, and aberrant cell proliferation, fostering a tumor-permissive microenvironment (84).

Two distinct macrophage subpopulations have been identified within the pleural cavity in murine models: large pleural macrophages and small pleural macrophages. The formers, the so-called tissue-resident population, are characterized by a decline that occurs in a few weeks after the beginning of carcinogenesis; on contrary, the smaller ones differentiate from circulating monocytes into M2-like macrophages, an immunosuppressive phenotype, expanding in TME soon after carcinogenesis establishment, becoming a stable population (85, 86).

M2-like macrophages, expressing CD163 and CD206, secrete IL-10, TGFβ alongside immunosuppressive mediators which promote aberrant cell proliferation, angiogenesis, cell mobility, fibrosis and metastatic processes (20, 84). Furthermore, a crosstalk between M2 macrophages and PM cells reinforces the immunosuppressive niche and the macrophage phenotype leading to enhanced prostaglandin E2 secretion, poor antigen presentation, and suppressed T-cell response (**Figure 3**) (84, 85, 87). In contrast, M1-like macrophages show pro-inflammatory and antitumor activity through the release of TNFα, IL-12, and NO (14, 88).

Several signaling pathways orchestrate TAM polarization in PM, including the CSF-1/CSF1R, STAT3, PI3K/AKT, and NF-κB axes (17, 20, 89). Hypoxia and mesothelial-derived factors such as HMGB1, VEGF, and CXCL12 further sustain macrophage recruitment and M2 polarization (16, 90). Bidirectional communications between mesothelial cells and macrophages, mediated by cytokines, extracellular vesicles, and metabolic exchange, reinforce an immunosuppressive niche that impedes T-cell infiltration limiting the efficacy of immunotherapies (86, 89, 90). Clinically, high infiltration of CD163^+^/CD206^+^ M2 macrophages correlates with poor OS and reduced response to ICIs (29, 85). Therapeutic strategies targeting TAMs, such as CSF1R inhibition, CD47 blockade, or M1 reprogramming, are showing promising results in preclinical and early translational studies (20, 90). Collectively, these findings underscore TAMs as central players in the immunopathology of PM and highlight their potential as both prognostic biomarkers and therapeutic targets.

## 3 Angiogenesis

Angiogenesis, the process by which new blood vessels form from the pre-existing vessels, represents a critical relevant step in the progression, growth, and metastasis of PM (91) (**Figure 3**). As a highly aggressive neoplasm arising from the mesothelial pleural lining PM requires an adequate blood supply to sustain continuous growth beyond a small size. In addition to supplying the tumor with oxygen and nutrients, the newly formed dense, disordered vascular network allows for the removal of metabolic waste products and provides routes for metastatic dissemination (91).

The angiogenic switch in PM is mainly a result of an imbalance of pro-angiogenic and anti-angiogenic factors within the TME. In particular, malignant mesothelial cells overly secrete proangiogenic factors, especially Vascular Endothelial Growth Factor A (VEGF-A), but also Fibroblast Growth Factor 2 (FGF2), Platelet-Derived Growth Factor (PDGF), and cytokines such as Interleukin-8 (IL-8) (92). Among the angiogenesis mediators in PM, VEGF, more precisely VEGF-A, is the most important and extensively studied molecule (93). This factor acts in a paracrine way on endothelial cells, inducing their proliferation, migration, and the formation of new vessels. Moreover, VEGF may also act as an autocrine growth factor, promoting the survival and proliferation of the mesothelioma cells themselves by binding to its receptors (VEGFR-1 and VEGFR-2) on their surface. Besides VEGF-A, other key growth factors, including basic Fibroblast Growth Factors 2(FGF2), and members of the Platelet-Derived Growth Factors (PDGF) family, are upregulated in PM and contribute to the angiogenic cascade. These factors often exert their effects in concert with VEGF-A, modulating the survival of Endothelial Cells (ECs) and recruitment of pericytes (94).

Specifically, Angiopoietin-1 (Ang-1) and Angiopoietin-2 (Ang-2), which bind to the Tie-2 receptor found mainly on ECs, are known as the critical regulators for vessel maturation and destabilization. Their imbalance brings about the abnormal structure of the tumor vasculature (95).

### 3.1 Roles of different TME cells in angiogenesis

PM cells are the initiating cells that drive the “angiogenic switch”, changing the state of the tissue from a resting to a highly vascular state. Notably, the cells of PM origin may co-express VEGFR-1 and VEGFR-2, which enable VEGF to serve as an autocrine growth factor that promotes proliferation, survival, and decreases the rate of apoptosis, irrespective of the role that is attributed to angiogenesis (96).

Endothelial cells (ECs) are the direct structural components and primary effectors of neovascularization, responding to the cues of TME. ECs, characterized by CD31 and CD34, are induced by paracrine VEGF to proliferate and migrate. VEGF binding to VEGFR-2 on the EC surface triggers the intracellular signaling pathway responsible for these functions (97).

Activated ECs release proteases, namely Matrix Metalloproteinases (MMPs), to penetrate through the basement membrane and then organize and form lumenized vascular tubes. The ensuing molecularly-assembled PM vasculature is typically structurally and functionally abnormal—tortuous, leaky, and disorganized. This abnormality results in Malignant Pleural Effusion (MPE) in which excess VEGF-mediated high vascular permeability causes plasma leakage, favoring the accumulation of protein-rich MPE, a leading clinical feature (98) (**Figure 3**).

Beyond the ECs, also TAMs, are actively recruited into the TME and play a key role in the activation of the angiogenic and immunosuppressive milieu. TAMs represent an important secondary source of pro-angiogenic factors, including VEGF-A, TNF-α, and Angiopoietin-2 (Ang-2). They also release substantial levels of MMPs and cathepsins, which facilitate the degradation of the extracellular matrix (ECM) creating a permissive niche that supports EC migration (85).

Importantly, TAMs have been implicated in mediating resistance against anti-VEGF therapy. By sustaining VEGF and other growth factor release, or by rerouting the TME to other pro-angiogenic pathways, they provide a mechanism of vascular escape when the VEGF pathway is blocked.

TME-activated fibroblasts, normally referred to as Mesothelioma-Associated Fibroblasts, produce structural support and critical paracrine signals.

Cancer-Associated Fibroblasts (CAFs) are the primary cells responsible for the dense desmoplastic stroma, a hallmark of most PMs. They produce abundant ECM proteins (*e.g.*, collagen), which not only create a rigid environment conducive to cell invasion but also sequester and release stored growth factors, which influence EC behavior. Indeed, CAFs release a number of soluble factors, including Hepatocyte Growth Factor (HGF) and PDGF, that induce proliferation and survival of PM cells and ECs. Such molecular and structural support is significant in stabilizing the tumor niche (99).

In addition, mural cells, including pericytes and smooth muscle elements, play a critical role in blood vessel maturation and function. Pericytes generally cover ECs and stabilize the vessel wall by way of the Angiopoietin-1 (Ang-1)/Tie-2 signaling axis. In PM, this interaction is frequently disrupted and causes defective pericyte coating. This immaturity is added to by an abnormally high expression of Angiopoietin-2 (Ang-2), which destabilizes the vessel wall, playing a key role in leaky phenotype and pathogenesis of MPE (100).

## 4 Molecular signatures as surrogate of the TME composition

TME composition has historically been investigated by immunohistochemistry (11); however, due to its two-dimensional nature and the limited number of potentially detectable markers, this approach has often failed to capture the marked intratumoral heterogeneity of the PM (101, 102). Consequently, over the last decade, substantial efforts have been made to indirectly characterize the PM TME by employing different multi-omics approaches (**Figure 4**). These technologies have enabled mapping of cellular composition and functional states within the TME (103–110), leading to the identification of novel immune and molecular signatures with potential predictive value for immunotherapy response.

**Figure 4:**
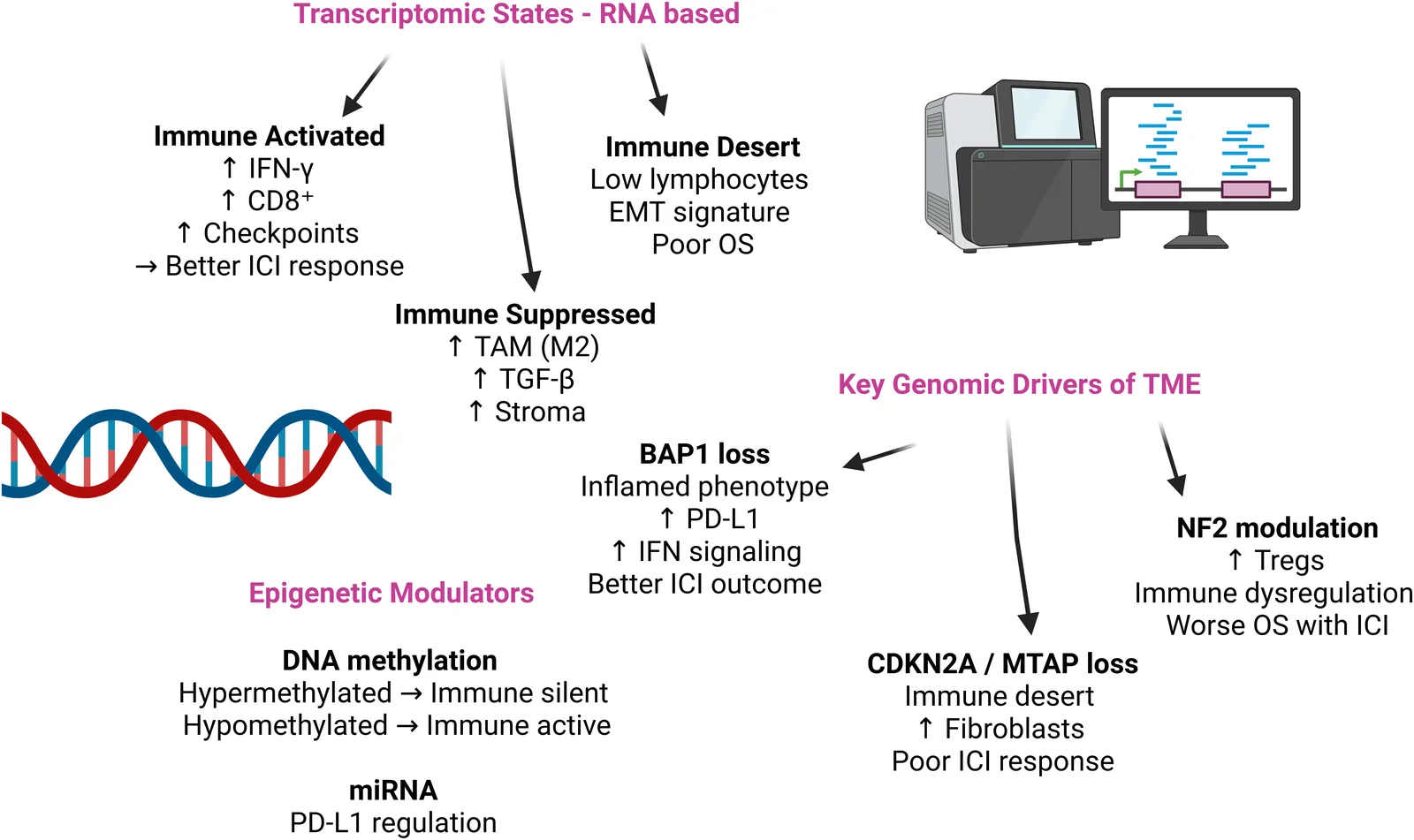
Molecular signatures defining the TME composition. Transcriptomic, epigenetic, and genomic alterations define distinct immune phenotypes, ranging from immune-activated to immune-desert states, thereby influencing the response to immunotherapy.

### 4.1 RNA-based signatures

RNA sequencing through computational deconvolution algorithms (111, 112) remains one of the most widely used methods to infer immune cell infiltration within PM, revealing the complexity of its TME. Indeed, despite the rarity of PM, an increasing number of transcriptomic analyses have been conducted in recent years, leading to the identification of immune-related gene signatures with prognostic and predictive relevance. For example, Yang et al., using the expression profile datasets of 330 PM samples, defined three immune-related subtypes named immune-activated, immune-suppressed, and non-immune tumors. The immune-activated subtype was characterized by high IFN-γ signaling, increased lymphocyte infiltration, marked TCR/BCR repertoire variability, together with the expression of IC molecules, and was associated with a better response to anti-PD-1 therapy. In contrast, the immune-suppressed subtype was enriched in stromal components, myeloid cell populations, and immunosuppressive TAMs, and exhibited increased activation of TGF-β–related signaling (113). Similarly, Alay and colleagues (2021) applied transcriptomic deconvolution to 516 PMs from seven RNA-profiling datasets to identify clinically relevant immune-cell fractions (114). Patients were stratified into three immune groups based on tumor T-helper 2 (Th2) and cytotoxic T-cell abundance. The IG1 group, which comprised more than 50% of the analyzed tumors, exhibited the shortest OS. In contrast, the IG3 group, accounting for only 8.5% of tumors, was characterized by high cytotoxic T-cell and low Th2 infiltration, together with upregulation of inflammatory and immune-related pathways and was associated with a more favorable prognosis. More recently, Behrouzfar et al. proposed a restricted six-gene signature (*IRF9, USP18, COL4A1, ITGA5, FN1, ITGB1*), derived from RNA-seq data initially identified in mouse models and subsequently refined and validated in large human PM cohorts, which identified tumors with immune-related phenotypes potentially responsive to immunotherapy (115). A recent blinded RNA-seq analysis was conducted within the phase III CONFIRM trial to develop a predictive model for response to anti–PD-1–based therapy. The study demonstrated that a pro-inflammatory gene signature can accurately predict response to nivolumab in patients with relapsed PM. In particular, responding tumors exhibited significantly higher expression of interleukins such as *IL17C, IL23A*, and IL24, together with chemokines including *CCL5, CCL19, and CXCL14*. Conversely, nivolumab-resistant tumors were enriched for epithelial-mesenchymal transition and mitosis-related signatures, with the mesenchymal transition being significantly associated with shorter OS (109). However, the clinical translation of bulk transcriptomic signatures in PM remains strongly challenged by the high degree of intra-tumoral heterogeneity. This limitation had already been highlighted by Meiller and colleagues in 2021, who, using multi-omic approaches including RNA sequencing of tumor samples taken from multiple sites from 16 patients with PM, demonstrated that the immune landscape can vary considerably within the same individual, with some tumors switching from ‘hot’ (high immune infiltration) to ‘cold’ (low immune infiltration) phenotypes across different anatomical sites (103).

To overcome these limitations, advanced RNA-based technologies, such as spatial transcriptomics (ST), have recently been applied to obtain a more refined characterization of the TME of PM, exploiting their ability to preserve tissue architecture while identifying spatially interacting cell populations (116–120). Among these studies, Torricelli et al. in 2023, using the GeoMx Digital Spatial Profiler to analyze 139 regions in eight patients with biphasic PM, demonstrated that epithelioid-sarcomatoid transition is driven by specific spatial niches (119). Their findings suggest that asbestos-induced inflammation creates a pro-tumorigenic system characterized by a reorganization of immune cell infiltration during tumor transition. Granulocyte populations, along with CD4^+^ and CD8^+^ T lymphocytes and B and NK cells, were predominantly observed in the sarcomatoid compartment, whereas Tregs and mast cells were enriched in the epithelioid counterpart. Similarly, Shek et al. through the Visium platform showed that immune cell infiltration is not stochastic, but follows specific patterns correlated with distinct tumor regions (120). A further study demonstrating this dynamic nature of PM heterogeneity was conducted by Severson and colleagues, who, by integrating bulk RNA sequencing, single-cell RNA sequencing (scRNA-seq), and ST at multiple pathological sites from 40 PM patients, identified a novel ‘uncommitted’ tumor cell state enriched in biphasic PM (116). This condition was also associated with a poor prognosis, particularly in epithelioid PM, underlining the prognostic value of tumor cell plasticity analyzed in their spatial context within the tumor.

Beyond spatial approaches, scRNA-seq has emerged as a powerful tool to resolve the cellular heterogeneity of the PM TME at single-cell resolution (121), enabling the identification of multiple PM molecular signatures with potential clinical relevance for treatment personalization (68, 104, 116, 122–125). Among these studies, one of the most comprehensive integrations of scRNA-seq of tumor and Peripheral Blood Mononuclear Cells (PBMC), identified 54 gene expression programs, generating a detailed cellular atlas of the PM TME (68). In addition, the authors also developed a single-cell sarcomatoid score (scScore) capable of capturing the gradient of sarcomatoid differentiation, a feature already associated with poor prognosis (106). Notably, scScore-high/sarcomatoid tumors showed malignant programs related to cell proliferation, migration, and displayed greater infiltration of exhausted T cells, together with Treg cells, CD8^+^ effector populations, and CXCL9+ macrophages. The authors also identified the NKG2A:HLA-E inhibitory axis between NK and tumor cells, indicating that blocking this checkpoint substantially increases NK cell cytotoxicity, offering a novel therapeutic strategy, particularly for epithelioid PM (68). In this context, Chin et al. also reported a longitudinal multi-omic analysis combining whole-blood RNA sequencing, scRNA sequencing, and single-cell T cell receptor sequencing (TCR-seq) of PBMCs, alongside tumor transcriptomic profiling, in 54 patients treated with chemoimmunotherapy within a single-arm phase II trial (126). The study identified an expansion of circulating CD8^+^ effector memory T cells with stem-like features in responding patients. Moreover, integrating dynamic PBMC immune signatures with baseline tumor transcriptomic data improved the accuracy of treatment response prediction. Recent scRNA-seq studies have highlighted the cellular heterogeneity and clinical relevance of malignant PM. Alay et al. identified three recurrent tumor cell states (stem-like, epithelial-like, and mesenchymal-like) with variable spatial distribution across tumor sites, and showed that mesenchymal-like signatures were associated with reduced sensitivity to standard chemotherapy, whereas stem-like programs suggested potential responsiveness to anti-angiogenic therapies (123). Consistently, Liu and colleagues defined three PM molecular subtypes with distinct prognostic and predictive implications using independent scRNA-seq datasets (124). Another relevant application of scRNA-seq *in vivo* model was conducted by Wu and colleagues to dissect the heterogeneity and functional states of TAMs in PM (85), revealing that small peritoneal/pleural macrophages preferentially exhibit an M2-like phenotype activating pro-tumorigenic programs like *KRAS* and TNF-α/NFkB signaling, whereas tissue-resident large peritoneal/pleural macrophages are linked to antitumor immune responses (IFN-γ).

Another complementary scRNA-seq study generated the first single-cell atlas of the human parietal pleura, revealing unprecedented stromal heterogeneity and highlighting a central role for TGF-β signaling in mesothelial-to-mesenchymal transdifferentiation (127).

### 4.2 Epigenome/genome-based signatures

Beyond RNA expression profiling, epigenetic alterations that modulate gene regulation have also been implicated in shaping the TME and have therefore been investigated for the development of prognostic and predictive signatures. Among epigenetic mechanisms of gene silencing, microRNAs (miRNAs) play a key role in regulating the expression of target genes involved in immune evasion. For instance, different miRNAs have been shown to directly target IC molecules such as PD-L1 (128, 129), thereby influencing tumor–immune interactions and potentially modulating the response to immunotherapy. In addition, multiple miRNA-based signatures have been associated with inflammatory processes within the TME correlating with the patient outcome (130–132). For instance, a four-miRNA signature comprising miR-181a-2-3p, miR-491-5p, miR-503-5p, and miR-3934-5p has been identified and shown to stratify PM patients at high risk (132). Importantly, analysis of the relationship between the expression of miRNA target genes, such as *KIF23* and *VPS37B*, and immune cell infiltration revealed significant correlations with CD4^+^ effector memory T cells, B cells, neutrophils, macrophages, and MDSCs. Further association analyses demonstrated a significant reduction of activated NK cells in the VPS37B-high expression group (p < 0.05), suggesting that VPS37B may influence NK-cell activity and, consequently, PM progression. In addition to small RNAs, long non-coding RNAs (lncRNAs), particularly those associated with glycolytic pathways, have been investigated to elucidate the relationship between this metabolic reprogramming and immune regulation. Notably, a glycolysis-related lncRNA signature (cluster 3) identified a subset of PM patients with poor clinical outcomes, characterized by high immune infiltration, pronounced activation of epithelial–mesenchymal transition programs, and elevated expression of IC molecules, including PD-1 and CTLA-4 (133). Another integrative approach combined mRNA, miRNA, and lncRNA data to construct a competing endogenous RNA network, identifying key survival-related RNAs (73 mRNAs, 13 miRNAs, and 26 lncRNAs) and selecting seven mRNAs and two lncRNAs for prognostic modeling. Immune infiltration analysis using the CIBERSORT algorithm revealed that the high-risk group patients were enriched in neutrophils, activated NK cells, and γδ T cells, whereas resting and activated dendritic cells, naïve B cells, M0 macrophages, monocytes, and naïve CD4 T cells were reduced (134).

In the epigenetic context, different DNA-methylation patterns have been described able to distinguish immune-active, hypomethylated tumors from immune-silent, hypermethylated ones, suggesting that methylation can predict immunogenicity (135, 136) also in the PM (137, 138). Indeed, different studies demonstrated that DNA methylation profiles in PM may strongly influence the tumor’s immune phenotype and thus its responsiveness to ICI (104, 125, 138). For example, Lofiego et al. in 2025 showed that PM cell lines can be stratified into two epigenetic classes, hypermethylated and hypomethylated, based on their CpG island methylator phenotype (CIMP) score, independently of histological subtype. The hypermethylated group displayed transcriptional silencing of immune-related pathways, including genes involved in antigen presentation, interferon signaling, and T-cell–inflamed signatures. Notably, treatment with the DNA hypomethylating agent such as the guadecitabine was able to reprogram these cells toward a more immunologically permissive transcriptional state, providing a strong rationale for evaluating epigenetic strategies in combination with ICIs in clinical settings (138). Recently, data from the NIBIT-EPI-MESO clinical trial, identified four distinct methylation subsets (i.e., demethylated, low, intermediate and CIMP) capable of predicting both response and OS following ICI-based therapy, independently of tumor histology (139). A multi-omic analysis integrating transcriptomic and epigenetic data generated a risk score showing that high-risk patients have a high TMB, which is nonetheless associated with a better response to ICIs; in addition, the risk groups showed distinct miRNA expression (mir-101–2, mir-101–1, mir-10a, and mir-107) as well as two DNA methylation subtypes mainly affecting metabolic pathways, together with a distinct immune infiltration; indeed, low-risk patients generally exhibited reduced infiltration of CD4^+^ Th2 T cells and plasma B cells, both linked to a more favorable prognosis, while showing higher infiltration of M2 macrophages (104). A similar multi-omics approach was employed to investigate the role of HLA-C, a class I major histocompatibility complex molecule, in immune modulation and PM progression (125). HLA-C expression was predominantly detected in macrophages, NK and T cells, within the TME. High HLA-C expression in immune cells was associated with a favorable prognosis, suggesting enhanced anti-tumor immune activity. Moreover, DNA methylation analysis identified 46 CpG methylation sites within the HLA-C locus. Hypermethylation of 12 out of 46 CpG sites (cg01006124, cg02827714, cg03216697, cg09970392, cg14931776, cg16097079, cg17096289, cg17974398, cg18020334, cg18747378, cg18785300, and cg24877963) was significantly associated with a worse clinical outcome, indicating that epigenetic silencing of HLA-C may impair immune surveillance and promote disease progression. In another relevant study, Jurmeister and colleagues evaluated the diagnostic value of genome-wide DNA methylation profiling combined with machine learning approaches to accurately distinguish PM from benign pleural conditions In addition, immune cell deconvolution analyses revealed a distinct TME in PM, characterized by higher proportions of Tregs and endothelial cells in tumor specimens, whereas chronic pleuritis exhibited a more heterogeneous inflammatory infiltrate, including macrophages, NK, and B cells.

Finally, genomic alterations have also been shown to correlate with the TME status and response to immunotherapy (16, 29, 110, 118, 140). Notably, a recent study by Torricelli et al. performed a phenogenomic analysis integrating whole-exome sequencing data with imaging mass cytometry, demonstrating the potential of TMB as a predictive biomarker. In particular, tumors with low TMB were associated with improved clinical outcomes and a higher degree of immune cell infiltration, especially macrophages and CD4^+^ T lymphocytes (29). In addition to the aggregate genome data derived from TMB, single-gene alterations can in turn represent key determinants of the TME state. For instance, *BAP1* tumor suppressor gene, which encodes a nuclear deubiquitinase pivotal for chromatin remodeling, DNA repair, and cell death regulation (141, 142), is frequently altered somatically in PM patients (143), while approximately 1-5% of individuals harbor germline alterations that confer a hereditary predisposition to cancer onset (140, 144, 145). *BAP1* alterations, which result in the loss of protein function, are frequently observed in immune-activated PM subtypes and are generally associated with a favorable outcome to ICIs (30, 110, 113, 143). Notably, this *BAP1*-deficient status correlates with a pro-inflammatory transcriptional phenotype, including interferon response, activating DCs, and IC expression (30, 110, 143). At the cellular level, the *BAP1*-deficient TME is characterized by an increased abundance of M2-polarized macrophages and precursor exhausted CD8^+^ T cells (PD-1^+^/TCF1^+^), alongside a reduction in MDSCs (30). Similarly, alterations in the neurofibromatosis type 2 (*NF2*) gene, a key regulator of the Hippo signaling pathway, have been associated with differences in the TME composition (146). In one of the first studies, Carlos Orozco-Castaño et al., applying a deconvolution algorithm (xCell) observed a significant increase in basophils, naïve B cells, and stromal cells (pericytes) in patients harboring *NF2* mutations (146). A more recent study further reported immune enrichment in *NF2*-mutant tumors using a deconvolution-based approach, although the increase in T cells (CD4^+^ and CD8^+^) did not reach significance (110). These findings were further confirmed by tumor tissue immunofluorescence analyses, which demonstrated an increased proportion of Tregs and cytotoxic T lymphocytes (p-value<0.03). However, the survival analysis on a retrospective data set of PM patients treated with ipilimumab plus nivolumab showed a two-fold shorter OS in patients harboring *NF2* mutations compared to wild-type ones (21 *vs* 42 months, p-value=0.006), potentially due to immunosuppressive counterparts such as Treg cells. Correlations between genomic alterations and the TME have also been explored for other genes frequently altered in PM, including cyclin-dependent kinase inhibitor 2A and 2B (*CDKN2A/B*) and methylthioadenosine phosphorylase (*MTAP*) (110). Alterations in these genes are consistently associated with a global reduction in immune cell infiltration, encompassing T cells, B cells, and NK cells, along with a concomitant increase in fibroblast abundance, resulting in a TME predominantly characterized by an immune-desert phenotype. In line with this immunologically cold microenvironment, patients harboring these alterations experienced worse clinical outcomes following ipilimumab plus nivolumab. Specifically, individuals with *CDKN2A* alterations showed a shorter median PFS (6.7 *vs* 10.6 months, p-value = 0.11) and a significantly reduced median OS (14 vs 42 months, p-value = 0.0003). Similarly, patients with *MTAP* loss exhibited a shorter median PFS (2.6 *vs* 5.6 months), and OS (median 30.5 *vs* not reached).

## 5 Therapeutic exploitation of TME in PM

### 5.1 Angiogenesis inhibitors

As previously detailed, angiogenesis is a rational target in PM. So far, several clinical trials have explored the role of angiogenesis inhibitors (both small tyrosine kinase inhibitors and monoclonal antibodies) as single agents or in combination with other agents in patients with PM.

#### 5.1.1 Small tyrosine kinase inhibitors as single agents or in association with chemotherapy

To date, a large number of small tyrosine kinase inhibitors, including sunitinib, dovitinib, pazopanib, cediranib, and nindetanib, have been tested in PM patients. Sunitinib is a TKI that exhibits a broad spectrum of tyrosine kinase receptor inhibition (VEGFR type 1 and 2, platelet-derived growth factor receptors PDGFR-alpha and PDGFR-beta; stem cell factor KIT; FMS-like tyrosine kinase 3-FLT3; RET and the receptor of macrophage-colony stimulating factor-CSF1R) (147) has been tested as a single agent in several phase II clinical trials. In the first trial, sunitinib was tested both in previously treated (cohort 1) and untreated PM (cohort 2) (148). The results of this trial were disappointing as only one objective response was reported in cohort 1 and median PFS and OS were 2.8 and 8.3 months in cohort 1, and 2.7 and 6.7 months in cohort 2, respectively. The second trial assessed the activity of single agent sunitinib at the same dose and schedule as the previous, in platinum/antimetabolite pretreated patients. In this trial, the primary endpoint was met as 12% of patients had a RECIST response. Median OS was 6.1 months for all the cohort. Dovitinib, a multi-kinase inhibitor with a broader spectrum of action encompassing FGFR1 in addition to VEGFR, KIT, FLT3 was also tested in a phase II trial (149) in relapsed PM. Although the trial met its primary endpoint, it was halted due to poor tolerability and the occurrence of early progressions. Furthermore, pazopanib was tested in a phase II clinical trial in pretreated PM. In this trial, pazopanib did not meet the prespecified primary endpoint and it was also associated with a high rate of G3–4 toxicities (150). Cediranib, a TKI with a narrower spectrum of activity, was evaluated both as a single agent and in combination with chemotherapy in PM patients. As a single agent, phase II trials (151, 152) demonstrated an underwhelming activity of cediranib, with an objective response rate of less than 10% and a high rate of grade 3–4 toxicity. Conversely, an improvement in response rate and PFS was observed in patients treated with the combination of cediranib, cisplatin, and pemetrexed in a phase II trial (153). Nevertheless, the small improvement in OS and the associated high rate of grade 3–4 toxicity precluded further development of the combination. A further exploration of the activity of small tyrosine kinase inhibitors in PM was made with nintedanib, a broad spectrum TKI was tested in association with standard chemotherapy in phase II/III studies. Despite the promising results of the phase II part showing a high objective response rate and prolonged PFS (154) compared to standard first line chemotherapy, the phase III part of the study did not confirm an improvement in PFS, and thus the combination wasn’t further explored (155). Finally, a phase II study tested nintedanib as a single agent in relapsed PM (156), but the results were dismal as no objective responses were observed.

#### 5.1.2 Combination of anti-VEGF and anti-VEGFR antibodies with chemotherapy

The anti-VEGF-A antibody bevacizumab has been tested in association with standard first line chemotherapy in the open label phase III IFCT (Intergroup Francaise de Cancerologie Thoracique) MAPS trial (157). In this study, patients with unresectable PM were randomly assigned to receive bevacizumab plus cisplatin/pemetrexed for six courses followed by maintenance bevacizumab versus cisplatin/pemetrexed for six courses. The primary outcome of the trial was the prolongation of the OS. Notably, the median OS proved to be longer in the combination arm (18.8 months; 95% CI 15.9-22.6) than in the standard arm (16.1 months; 95% CI 14-17.9), with a Hazard Ratio of 0.77 (p-value=0.0167) (157). Ramucirumab, an anti-VEGFR2 monoclonal antibody, has also been tested in combination with gemcitabine in phase II RAMES trial. In this trial patients were randomized to receive either gemcitabine plus ramucirumab or gemcitabine alone and the primary outcome was OS, significatively longer in the ramucirumab arm (13.8 months, 70% CI 12.7-14.4) than in placebo arm (7.5 months; 70% CI 6.9-8.9) with a Hazard Ratio of 0.7 (70% CI 0.59-0.85; p-value=0.028) (158).

#### 5.1.3 Combination of antiangiogenic drugs with ICIs

The combination of pembrolizumab and nintedanib (tyrosine Kinase Inhibitor targeting VEGFR) has been explored in the mesothelioma arm of the Phase IB trial PEMBIB [NCT02856425] (159). Despite testing, the efficacy of the combination was not the main endpoint of the trial, the combination was found to be intriguingly active. Indeed, the median PFS was 6.2 months (95% CI, 3–8.7). At database lock, the median OS was 14.1 months (95% CI, 9.89–not reached). Furthermore, in patients achieving durable clinical benefit (*i.e.*, a disease stabilization lasting at least 6 months), the median OS was 23.6 months. Promising results were also shown with the combination of pembrolizumab and lenvantinib in treatment of refractory PM in the PEMMELA trial (160). In this phase II trial, 38 patients affected by PM in the 2nd or 3rd line were treated with the combination of pembrolizumab (200 mg every three weeks) and lenvantinib (20 mg daily). The trial was positive as 58% of enrolled patients achieved a partial response, but serious adverse events were frequent (26%, with one toxic death due to myocardial infarction), as the need for dose reduction. The phase III BEAT meso trial (161), explored the effect on OS of the combination of atezolizumab plus cisplatin, pemetrexed, and bevacizumab versus platinum plus pemetrexed and bevacizumab in treatment naïve PM. The trial was formally negative as no improvement in OS in the intention to treat population (20.5 months versus 18.1 months; HR 0.84, 95% CI 0.66-1.06, P = 0.14), the primary endpoint of the trial, was demonstrated for the combination arm. Nevertheless, the addition of atezolizumab to standard chemotherapy plus bevacizumab significantly increased PFS (9.2 versus 7.6 months; HR = 0.72, 95% CI 0.59-0.89, p-value= 0.0021). The benefit of this combination was more pronounced in patients with non-epithelioid disease with an HR of 0.50, (95% CI 0.32-0.78, p-value = 0.002) for OS and 0.43 for PFS (95% CI 0.28-0.66, p-value < 0.001). Finally, a phase II trial investigated the combination of nivolumab and ramucirumab in relapsed pleural mesothelioma (162). The trial was negative, as ORR was 22.6% (95% CI: 9.6%-41.1%) in the whole population and 43% (95% CI: 10%-82%) in patients with non-epithelioid histology. PFS rate at 24 weeks was 32% (95% CI: 17%-51%). Median PFS was 4.2 months (95% CI: 1.9-6.4 mo). Median OS was 12.5 months (95% CI: 6.3-23.5 months). Also, in this trial, patients with non-epithelioid histology derived most benefit from the combination of treatment.

The role of antiangiogenic drugs in combination with ICIs is yet to be defined, and several trials are underway in which this combination is exploited. A phase II trial ongoing in Japan, PENINSULA [NCT06318286] is evaluating the activity of the combination of pembrolizumab, lenvatinib, and platinum-based chemotherapy in the first line in PM (**Table 3**). In PENINSULA, patients with unresectable PM and an adequate ECOG performance status, will undergo to induction treatment with low dose lenvatinib (8 mg daily) plus pembrolizumab and standard chemotherapy for 6 courses, followed by maintenance pembrolizumab and lenvatinib, up to 35 courses. The main endpoint of the trial will be Overall response rate, with PFS, OS and disease control rate as secondary endpoint (163). Similarly, further evidence of the efficacy of the combination of ICIs with anti-VEGFR TKI will derive from a phase II trial ongoing in China in which the combination of sintilimab, an anti-PD1 and anlotinib, a multi-target TKI with a broad therapeutic spectrum (VEGFR, FGFR, PDGFR and KIT) is explored in combination with standard first line chemotherapy [NCT05188859]. A single arm phase II trial exploring the same combination is ongoing in patients with malignant PM in China [NCT06543069]. In this context, bispecific antibodies, *i.e.*, monoclonal antibodies engineered to bind two different antigens or epitopes simultaneously, designed to target both PD1/PDL1 and angiogenesis, are currently tested in thoracic cancers including mesothelioma. In particular, bispecific antibodies represent an evolution beyond the co-administration of separate monoclonal antibodies. Although both strategies may inhibit the same signaling pathways, bispecific constructs integrate two binding specificities within a single molecule, allowing coordinated and simultaneous target engagement with shared pharmacokinetic properties, tissue distribution, and exposure (164, 165). Mechanistically, this may increase functional avidity when both targets are present within the same TME and may favor localized dual pathway modulation compared with the independent biodistribution of two separate antibodies. For example, PD-1/VEGF bispecific antibodies can simultaneously engage checkpoint-expressing immune cells and VEGF within the TME, thereby enhancing T-cell activity while counteracting VEGF-mediated immunosuppression, aberrant angiogenesis, and impaired immune cell trafficking. This cooperative binding behavior has been described for ivonescimab, which can form complexes with VEGF and display enhanced PD-1 binding avidity in the presence of VEGF, and similar principles may apply to other dual immune checkpoint/angiogenic pathway bispecific constructs, such as BNT327 (pumitamig) (166). Thus, in contrast to the simple pharmacological combination of two monoclonal antibodies, bispecific antibodies may provide spatially coordinated, target-enriched modulation of complementary immunosuppressive pathways within the TME. Based on this mechanistic rationale, different bispecific antibodies are currently being evaluated in mesothelioma. BNT327 (Pumitamig), a bispecific antibody targeting PD-1 and VEGF-A, has been recently evaluated in combination with standard platinum-based chemotherapy in a Phase II trial in both pleural and peritoneal mesothelioma. The results of this trial have been presented at the 2025 American Society of Clinical Oncology conference and were deemed positive as 43.5% of pleural and 75% of peritoneal mesothelioma patients achieved at least a partial response. Another phase II trial is testing the combination of cadonilimab (a bispecific anti-PD1/anti-CTLA-4 antibody) and bevacizumab with standard platinum pemetrexed chemotherapy, which is ongoing in first line in mesothelioma [NCT05930665]. In the same patient setting, ivonescimab is being tested as a single agent in a Phase II trial ongoing in France, Bi-MAPS[NCT06840834], and in combination with standard chemotherapy (either single agent gemcitabine, pemetrexed or vinorelbine) in refractory PM a phase II trial in China [NCT06875076].

**Table 3:** Ongoing clinical trials targeting TME in pleural mesothelioma.

Study ID	Phase	Disease setting	Immunological target	Investigational agent	Estimated enrollment	Primary endpoint	Trial location
NCT06318286 (PENINSULA) (163)	II	Pleural	PD-1/VEGFR	Pembrolizumab + lenvatinib + platinum-based chemotherapy	25	Response rate	Japan
NCT05188859	II	Pleural	PD-1/angiogenesis	Sintilimab + anlotinib + standard CT	29	Objective response rate	China
NCT06543069	II	Peritoneal	PD- 1/angiogenesis	Sintilimab + anlotinib + standard CT	28	Progression free survival	China
NCT05930665	II	Pleural and peritoneal	PD-1/CTLA-4/VEGF	Cadonilimab + bevacizumab + platinum/pemetrexed	38	Objective response rate	China
NCT05918107	II	Pleural and peritoneal	PD-1/angiogenesis	PM8002 (BNT 327- pumitamig) + chemotherapy	31	Coprimaries: Objective response rate; Treatment related adverse events	China
NCT06840834 (Bi-MAPS)	II	Pleural and peritoneal (> 2nd line)	PD-1/VEGF-A	Ivonescimab	38	12 weeks disease control rate	France
NCT06875076	II	Pleural and peritoneal (> 2nd line)	PD-1/VEGF-A	Ivonescimab	25	Objective response rate	China
NCT05304208 (ENSURE)	I	Pleural and Perioperative	DCs/antigen presenting cells	MesoPher	16	Number of patients who are alive and completed neo-adjuvant plus adjuvant Dendritic Cell Therapy and surgery at week 15 (+4 weeks) without treatment-related delay, persisting grade 3–4 toxicities or progression/relapse	Netherlands
NCT05765084 (Immuno-MESODEC)	I/II	Pleural	WT1-directed immunity/PD-L1	WT1 mRNA DC vaccine+ atezolizumab + chemotherapy	15	Coprimaries: Proportion of patients experiencing TRAEs; Number and grade of Aes and SAEs; proportion of patients who completed treatment schedule	Multinational
NCT07234058 (LAG-MAPS)	II	Pleural	PD-1/*LAG-3*	Cemiplimab + fianlimab + chemotherapy	126	6 months disease control rate	France
NCT06790706 (immunorare-5)	II	Peritoneal	PD-1/TIGIT	Domavanalimab/zimberalimab	27	24 weeks progression free survival rate	France
NCT06097728 (eVOLVE-Meso)	III	Pleural	PD-1/CTLA-4	Volrustomig + carboplatin + pemetrexed	863 (actual)	Overall Survival in the epithelioid histology patients	Multinational

### 5.2 Exploiting TME cells: innate immunity

#### 5.2.1 Monocytes and macrophages

Direct inoculation of innate immunity cells in the pleural space of PM patients has been tested in the past as a first attempt to use innate immunity against PM. In phase II trial, patients affected by stage IA, IB or IIA PM received weekly intrapleural infusions of autologous human activated macrophages (AM Phi) for 8 consecutive weeks (167), followed by an intrapleural IFNγ infusion three days after the macrophage infusion.

The main objective of the trial was the overall response rate. While the trial was deemed negative, as the overall response rate was 14% (11% in the intention to treat population), the patients achieving an objective response had durable responses, with one patient achieving disease stabilization, which lasted 67 months. Furthermore, ONCOS-102, a chimeric oncolytic adenovirus engineered to express human granulocyte-macrophage colony-stimulating factor, with proven *in vitro* cytolytic and immunostimulant activity, has been tested in PM. In a phase II trial, ONCOS 102 injected directly into the tumor in combination with standard platinum pemetrexed chemotherapy showed a numerically longer OS and enhanced tumor activation when compared with standard platinum pemetrexed therapy (168).

#### 5.2.2 Exploiting TME cells: dendritic cells and antigen-presenting cells

ABBV-428, a bispecific antibody binding to mesothelin expressed on cancer cells and to CD40 expressed on antigen-presenting cells, was tested in a Phase I trial in relapsed pleural mesothelioma and ovarian cancer. In this trial (169), no objective responses were observed, but long disease stabilization exceeding one year was found in three cases. Intriguingly, two of these patients experienced a disease response with an antiPD1 following ABBV-428 failure.

The exploitation of dendritic cells as antigen-presenting cells to enhance autologous immunity against PM also seemed like a rational strategy to treat mesothelioma. In the Phase II DENIM (DENdritic cells Immunotherapy for Mesothelioma) trial, Mesopher, an injection of DCs loaded with allogeneic tumor cell lysate, was tested versus best supportive care in PM patients achieving at least a disease stabilization with standard first line platinum/pemetrexed-based chemotherapy (170). The primary endpoint of the trial was OS and consolidation treatment with Mesopher did not improve the survival when compared to best supportive care (16.8 months; 95% CI 12.4-20.3; in the MesoPher group *versus* 18.3 months; 95% CI 14.3-21.9; in the best supportive care group; HR = 1.10; 95% CI 0.77-1.57; log-rank p-value=0.62). Considering previous experiences, treatment with DCs alone might seem like an underwhelming strategy to treat mesothelioma, at least in the maintenance setting. Investigation on this pathway is still ongoing, and MesoPher is currently being evaluated in the perioperative setting in a phase I trial ongoing in the Netherlands [NCT05304208]. In addition, emerging trials such as the Immuno-MESODEC (171) (NCT05765084) trial in which a WT1 mRNA-based DC vaccine is administered with standard chemotherapy and the anti-PDL1 atezolizumab are combining autologous DC vaccines and ICIs, to provide a synergistic immunotherapy combination.

### 5.3 Beyond PD-L1 and CTLA-4: other potentially actionable immune checkpoints

#### 5.3.1 LAG-3

The combination of ieramilimab (anti-LAG-3) and spartalizumab (anti-PD-1) demonstrated an encouraging clinical activity in patients affected by PM in a phase I clinical trial (172), leading to the development of a phase II trial in pretreated PM patients (173). Overall, 57 patients were enrolled in this phase II trial, of whom 41 were anti-PD-1/PD-L1 naïve and 16 anti-PD-1/PD-L1 pretreated. Overall response rate was 17% (2 complete responses and 5 partial responses) among naïve patients and 6% (1 partial response) in pretreated patients. Similarly, PFS was longer among anti-PD-1/PD-L1 naïve patients (5.5 months) than in pretreated patients (3.3 months). A further Phase II trial, exploring in first line the activity of the combination of cemiplimab (anti-PD-1) and fianlimab (anti-LAG-3) *versus* the combination of cemiplimab and standard first line chemotherapy, LAG-MAPS, is currently ongoing in France [NCT07234058].

#### 5.3.2 TIGIT

Few data are available regarding the activity of anti-TIGIT in PM. In the phase I trial AdvanTG-105, two patients affected by epithelioid PM were treated with the combination of tislelizumab (anti-PD1) and ociperlimab (anti-TIGIT) and achieved a partial response (174). Parallel studies conducted in murine models demonstrated greater efficacy of anti-TIGIT and anti-PD-1 compared with the other combinations tested (anti-PD-1 plus anti-CTLA-4, anti-PD-1 plus chemotherapy, and the triplet regimen). Currently, the combination of domavanalimab (an anti-TIGIT antibody) and zimberelimab (an anti-PD-1 antibody) is being tested in a multi arm phase II trial in which arm 1 will enroll patients with peritoneal mesothelioma [NCT06790706].

#### 5.3.3 VISTA

PM is the tumor with the highest expression of the immune checkpoint VISTA (64). Twelve patients affected by PM were treated with the oral anti-VISTA drug CA-170 in a phase Ib study. Although no objective responses were observed, stable disease was achieved in 7 patients. Patients treated with a higher dose of 1200 mg twice daily derived the most benefit from the treatment, achieving a median duration of disease control of four months. No new safety signals were identified, and drug-related adverse events were infrequent.

## 6 Discussion

TME plays a major role in PM pathogenesis, mainly through the induction of an immunosuppressive milieu in which tumor cells proliferate. This pathogenic alteration might conversely be exploited as a therapeutic target in PM.

Indeed, anti-PD-1, either in combination with anti-CTLA-4 or with chemotherapy, has recently become the therapeutic standard in PM, with a more pronounced benefit in non-epithelioid histology. However, some concerns regarding the real benefit of ICI in PM, both in the first and in further therapeutic lines, have been raised so far. First, a thorough literature review and methodological analysis of available evidence (both randomized and real life) of combination ICI treatment in PM, highlights methodological, toxicity and economic issues in using ICI in all-comers PM patients, and suggests using these expensive drugs should be limited to the rarer and more aggressive non-epithelioid histology (175). Second, a comparison of OS in MAPS trial and Checkmate 743 trial reports no statistically significant difference in OS between the experimental arms of the two trials (HR 0.97 [95% CI, 0.79-1.20]; P = .79), with an OS benefit restricted to non-epithelioid mesothelioma in Checkmate 743 (176). Finally, clinical trials with anti-PD1 monotherapy in further lines in PMs have proved discording results, as pembrolizumab (177) revealed a similar efficacy to classic single agent drugs in second line, while only nivolumab proved to prolong OS over placebo in the CONFIRM trial (178). This led to methodological criticism of the CONFIRM trial (179), because of the underwhelming experimental arm.

Despite acknowledging all the previous limitations to ICI use in PM, these compounds are a mainstay of mesothelioma treatment, especially in non-epithelioid subtypes. While research on predictive biomarkers is undergoing, a simple yet useful intervention to improve ICI efficacy in PM is to fully implement surgical biopsies for the diagnosis of PM in patients eligible for medical treatment, given the lower sensitivity of CT guided and medical thoracoscopy in detecting non-epithelioid disease when compared to surgical biopsies (180, 181). Indeed, TME differs substantially across PM histological subtypes, with epithelioid PM typically exhibiting a more immune-activated yet partially excluded milieu enriched in lymphoid populations, whereas non-epithelioid subtypes are characterized by a predominance of immunosuppressive myeloid cells and regulatory T cells, consistent with their more aggressive clinical behavior. These differences likely contribute to the distinct responses to immunotherapy observed across subtypes, with non-epithelioid mesotheliomas, despite their poorer prognosis, often deriving greater benefit from ICIs due to a more inflamed, albeit suppressive, TME, whereas epithelioid tumors may require combination strategies to overcome immune exclusion and enhance effective antitumor immunity.

CTLA-4 blockade, by enhancing priming and expansion of tumor-reactive T cells in lymphoid tissues, increases clonal diversity and reduces regulatory T-cell-mediated suppression, thereby potentially increasing the pool of effector T cells capable of infiltrating tumors (**Figure 5**). This mechanism is particularly relevant in mesothelioma, where TME is often characterized by profound immunosuppression driven by regulatory T cells, tumor-associated macrophages, and T-cell exhaustion. In more inflamed histological subtypes such as sarcomatoid PMs, CTLA-4 blockade may further amplify an already existing immune infiltrate, improving the likelihood of effective cytotoxic responses. Conversely, in more immune-excluded tumors, such as epithelioid mesothelioma, CTLA-4 blockade alone is often less effective because the primary limitation is not insufficient T-cell priming, but rather impaired trafficking and infiltration of effector cells into the tumor bed. Thus, while CTLA-4 blockade can broaden and diversify the antitumor T-cell repertoire across mesothelioma subtypes, its clinical impact in epithelioid disease could be maximized when paired with interventions that facilitate T-cell trafficking and relieve intratumoral suppression, rather than relying on priming effects alone. Therefore, future clinical studies should be addressed toward combination therapies integrating CTLA-4 blockade with compounds that promote T-cell infiltration into the TME.

**Figure 5:**
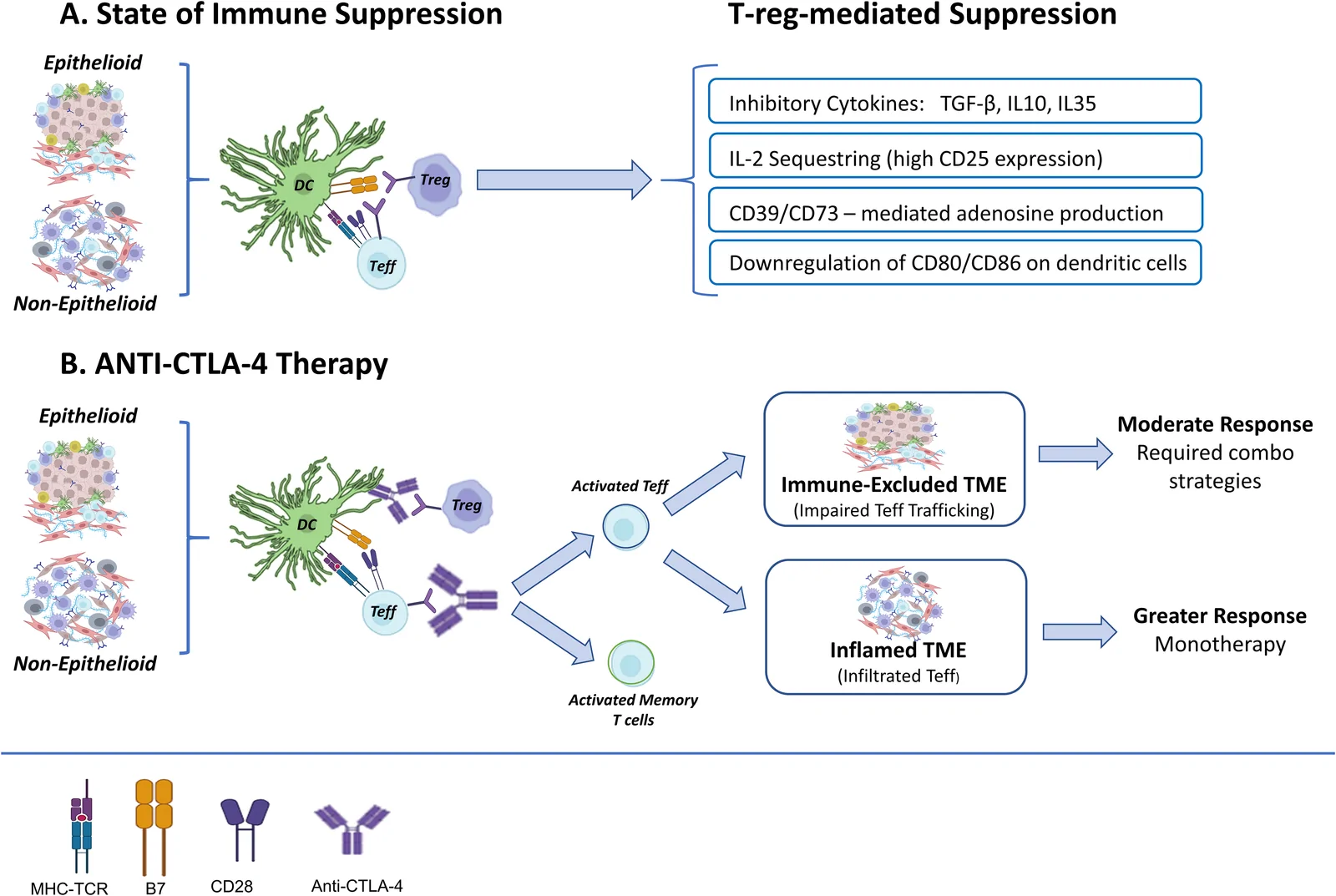
Schematic illustration explaining how CTLA-4-based immunotherapy may be effective in mesothelioma. **(A)** State of Immune Suppression: Tregs inhibit effector T cell (Teff) activation and promote tumor growth through I) inhibitory cytokines, II) IL-2 sequestration, III) adenosine production, and IV) downregulation of CD80/CD86 on dendritic cells (DC). **(B)** Anti-CTLA-4 blockade restores co-stimulation (CD80/CD86 to CD28), activating Teff and memory T cells. The clinical benefit depends on histology: immune-excluded epithelioid tumors show a moderate response, while inflamed non-epithelioid/sarcomatoid tumors derive a greater survival benefit.

Despite the advancement in novel therapeutic approaches in PM patients, the lack of robust and reliable predictive biomarkers for current treatments remains a major unmet clinical need. Indeed, unlike other thoracic malignancies, in which PD-L1 tumor expression is routinely used as a predictive biomarker of response to ICIs (182), the role of PD-L1 in PM remains controversial and affected by significant limitations (**Figure 6**). Clinical studies have demonstrated that PD-L1 expression is highly heterogeneous, both intra-tumors and among different histological subtypes. Moreover, PD-L1 status does not consistently correlate with clinical outcomes, as responses to ICIs may occur in both PD-L1–positive and PD-L1–negative patients (7, 53, 183) and the current available evidence remains conflicting (32, 184, 185). In addition, variability in detection assays, scoring methodologies, and positivity cut-off thresholds further limits the reproducibility and clinical applicability of this biomarker (186). Overall, PD-L1 expression captures only a limited dimension of the TME and is insufficient as a standalone predictive biomarker in PM. Similarly, TMB has been proposed as a potential predictor of response to ICIs, based on the rationale that a higher number of mutations per megabase may increase the generation of immunogenic neoantigens and enhance antitumor immune responses (187). However, TMB has not demonstrated significant predictive value in PM, likely due to the intrinsically low mutational burden that characterizes mesothelioma. Indeed, exploratory analyses from the CheckMate 743 trial did not show any significant association between high TMB and improved overall survival (53). This lack of predictive performance may also reflect technical limitations of current NGS approaches, which may fail to adequately capture the complex genomic architecture of PM, including chromosomal rearrangements that could potentially generate neoantigens (188).

**Figure 6:**
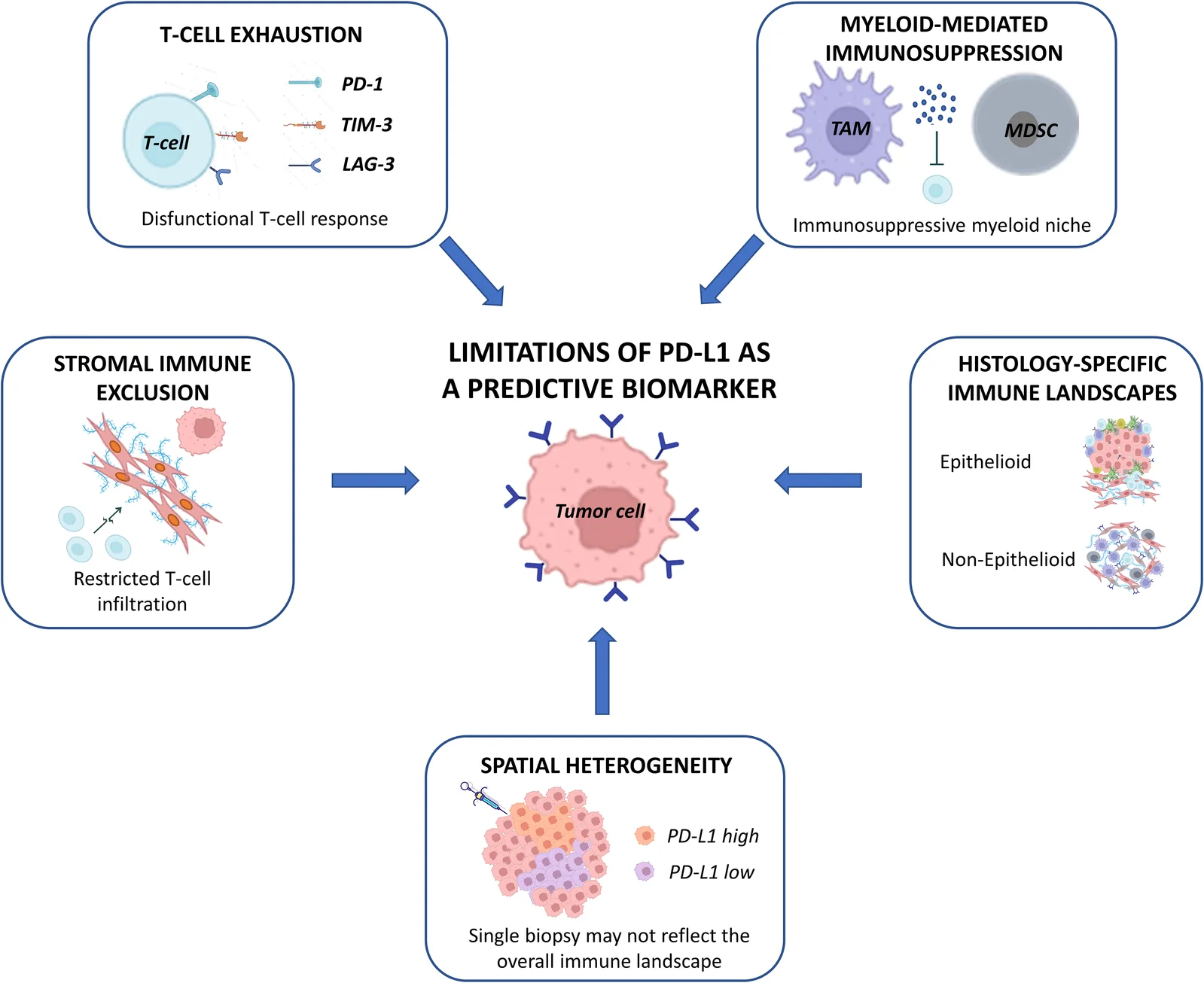
Schematic representation of the limitations of tumor PD-L1 as a standalone predictive biomarker in PM. The predictive relevance of tumor PD-L1 expression can be influenced by multiple features of the tumor microenvironment, including T-cell exhaustion, myeloid-mediated immunosuppression, stromal immune exclusion, spatial heterogeneity, and histology-specific immune landscapes, supporting the need for integrated biomarker strategies.

In contrast, more comprehensive immune profiling, particularly of T-cell exhaustion markers, may provide a more robust framework for identifying patients most likely to benefit from ICIs and for guiding the rational development of combination strategies. Importantly, greater efforts should be directed toward identifying patients who derive the greatest benefit from conventional therapies, including chemotherapy and multimodal approaches. These strategies should not be considered mutually exclusive, but rather complementary components of an integrated approach to optimize treatment selection. Within this framework, comprehensive profiling of the TME may serve a dual purpose: (i) to identify the subset of patients most likely to benefit from immunotherapy, and (ii) to elucidate mechanisms of resistance, thereby informing the development of more effective and potentially cost-efficient therapeutic combinations. In parallel, although the therapeutic exploitation of novel TME-related targets is actively ongoing, and early-phase trials are yielding encouraging results appropriate patient stratification remains a critical unmet need. In particular, strategies that move beyond PD-L1 expression or histology-based stratification alone are essential to maximize clinical outcomes in this difficult-to-treat disease.

In this regard, growing evidence have demonstrated that genomic, transcriptomic, miRNome, and methylation–based signatures can indirectly capture complementary dimensions of TME interactions in PM, collectively delineating complex immune infiltration partners with prognostic and/or potential predictive relevance. However, despite the growing number of these immune-related signatures, their translation into clinical practice remains limited. Indeed, most currently available signatures derive mainly from retrospective cohorts and/or heterogeneous datasets generated using different panels, sequencing platforms, and deep sequencing, resulting in limited reproducibility across studies. Furthermore, the rarity of PM strongly limits sample size, leading to insufficient statistical power and increasing the risk of model overfitting, particularly for the development of predictive rather than only prognostic biomarkers. On the contrary, signatures developed within prospective clinical trials may provide a more robust framework for biomarker development, thanks to standardized sample collection, homogeneous treatment exposure, as well as predefined clinical endpoints. In this context, a four-gene inflammatory expression signature combining *CD8A*, *STAT1*, *LAG3*, and *PD-L1*, designed to capture the impact of tumor microenvironment inflammation on clinical outcomes, emerged as a promising biomarker in the Phase III CheckMate 743 study (6). Specifically, a high score was associated with improved survival benefit with nivolumab plus ipilimumab (median OS of 21.8 months in the high-score group versus 16.8 months in the low-score group; HR 0.57), with corresponding 3-year OS rates of 35% and 15%, respectively. In contrast, no predictive effect was observed in the chemotherapy arm (median OS 11.6 *vs.* 15.2 months; HR 1.14). However, a further significant obstacle to the clinical application of these genome/transcriptome-based signatures in PM is related to its extreme spatial and intratumoral heterogeneity (101, 103, 189). This heterogeneity is not exclusively a technical confounding factor but reflects the biological property of the disease. Indeed, PM is characterized by the coexistence of distinct intra-tumoral immune niches, which often exhibit divergent immune compositions, stromal activation, and states of malignant cells reflecting the patient’s outcome. This spatial organization has clinically relevant implications for biomarker identification, mechanisms of treatment resistance, and consequently the response to immunotherapy. From a biomarker development perspective, spatial heterogeneity challenges the assumption that a single tumor biopsy can adequately capture the immune landscape of PM. Indeed, transcriptomic and genomic signatures derived from bulk samples inevitably average signals across spatially distinct regions, thereby masking localized immunoactive niches or, conversely, immunosuppressive compartments that could determine clinical outcome. Therefore, spatial heterogeneity may also explain resistance and unpredictable responses in PM as immune-reactive regions can coexist with immunosuppressive tumor areas. As a result, ICIs may restore antitumor immunity only in selected tumor regions, making treatment response dependent on the spatial balance of these different ecosystems.

ST is recently emerging as a key technology to overcome this limitation, preserving tissue architecture and enabling the identification of immuno-tumor interactions, although it remains confined to a limited two-dimensional tumor area. ScRNA sequencing also offers the advantage of analyzing cell diversity and functional states at high resolution, enabling the identification of rare but clinically relevant immune and tumor subpopulations. Similarly, epigenomic profiling, including DNA methylation-based classification, may provide a complementary layer of information able to capture both tumor lineage and immune contexture that might not fully extracted by transcriptomic analyses alone. Overall, nevertheless ST and single-cell approaches are generating unprecedented biological insights, they currently remain more suitable for research and specialized translational settings than for routine laboratory diagnostics. A critical next step is therefore to translate these high-dimensional multi-omic signatures into focused, standardized, and clinically feasible diagnostic assays. Although scRNA-seq, spatial transcriptomics, and genome-wide methylation profiling are powerful discovery platforms, their direct implementation in routine diagnostics is limited by cost, technical complexity, tissue requirements, turnaround time, and the need for dedicated bioinformatic infrastructure. These approaches may instead guide the selection of reduced biomarker sets that can be converted into FFPE-compatible RT-qPCR or targeted expression panels, multiplex immunofluorescence/immunohistochemistry assays, and targeted methylation tests. Such assays could enable the reproducible assessment of immune-inflammatory transcripts, spatially relevant cell-cell interactions, and PM-associated methylation classes while remaining compatible with standard pathology workflows. Importantly, applying these targeted assays across multiple tumor may help mitigate the impact of spatial heterogeneity and better capture the coexistence of immune-active and immunosuppressive niches. Another major open question regarding the immune signatures concerns how tumors shape their TME under therapeutic pressure, since most of them derive from diagnostic tumor samples and therefore fail to capture the dynamic evolution of the immune microenvironment during treatment. In this context, liquid biopsy–based immune signatures represent an attractive and minimally invasive alternative that may be repeated longitudinally, providing real-time insights into TME remodeling and resistance development during therapy. PE-TME composition inferring is of great potential interest in malignant PM, since clinical thoracentesis provides access to a relatively rich and accessible immune compartment. However, early evidence from PE–based TME characterization indicates that immune cell composition and functional states derived from PEs do not fully recapitulate intratumoral immune landscapes and may therefore lack robust prognostic or predictive value (25, 190). In this regard, a recent study has shown that macrophages exposed to PE-derived factors acquire a tumor-promoting M2-like phenotype and suppress T-cell proliferation (191). Consistently, macrophages isolated directly from PEs display an M2 phenotype and a negative correlation with T-cell abundance. In addition, a recent liquid biopsy-based study comparing T cells derived from PEs and matched PBMCs demonstrated that PE-derived T cells express higher levels of immune checkpoint molecules (*i.e.*, PD-1, LAG-3, and TIM-3), whereas PD-1 on CD4 T cells from PBMCs emerged as an independent prognostic factor for poor OS, suggesting that immune phenotypes captured in malignant PEs differ from those observed in circulation (192). Further supporting the clinical relevance of peripheral blood-based immune profiling, the 5-year update of the CheckMate 743 trial showed that higher baseline circulating M-MDSC levels were associated with poorer outcomes in patients treated with nivolumab plus ipilimumab, supporting the potential role of peripheral blood M-MDSC quantification as a promising, minimally invasive biomarker for patient stratification (13). Overall, these findings support the complementary use of PEs and peripheral blood as minimally invasive sources to capture distinct but interconnected aspects of the immune landscape in the PM.

A further potential strategy to overcome the previous limitations could be represented by radiomics. By extracting high-dimensional quantitative features from computed tomography (CT) images, radiomic approaches can theoretically capture the 3D-spatial and biological heterogeneity of the entire tumor in a minimally invasive manner. Notably, radiomics-based models have already shown promise in mapping tumor immune status and predicting treatment response in other chest malignancies (193, 194), supporting their potential applicability to PM.

In the near future, integrative strategies combining TME, spatially resolved tissue profiling, single-cell analyses, and longitudinal liquid biopsy data alongside radiomics will be essential for capturing immune heterogeneity and its temporal evolution, understand resistance mechanisms, and optimize immunotherapeutic strategies in pleural mesothelioma. To date, a signature potentially predictive to ICIs should integrate multiple complementary indicators, such as the TME inflammatory status, such as the four-gene signature, together with the methylation profile (i.e., demethylated, low, intermediate, and CIMP) (139).

Furthermore, large-scale, multi-institutional prospective studies with multi-regional sampling and longitudinal immune monitoring will be crucial to define reproducible and clinically actionable immune signatures for patient stratification and the identification of new targeted immune and tumor therapies to improve the treatment of this deadly disease.

## References

[1] Carbone M Adusumilli PS Alexander HR Baas P Bardelli F Bononi A . Mesothelioma: scientific clues for prevention, diagnosis, and therapy. CA Cancer J Clin. (2019) 69:402–29. doi: 10.3322/caac.21572 31283845 PMC8192079

[2] Fennell DA Sekido Y Baas P Husain AN Curioni-Fontecedro A Lim E . Pleural mesothelioma. Nat Rev Dis Primers. (2025) 11:56. doi: 10.1038/s41572-025-00640-3 40775245

[3] Lim E Waller D Lau K Steele J Pope A Ali C . Extended pleurectomy decortication and chemotherapy versus chemotherapy alone for pleural mesothelioma (MARS 2): a phase 3 randomised controlled trial. Lancet Respir Med. (2024) 12:457–66. doi: 10.1016/S2213-2600(24)00119-X 38740044 PMC11136673

[4] Ripley RT Adusumilli PS Bograd AJ Bölükbas S Bueno R Cameron RB . Going to MARS may shorten our patient’s survival. J Thorac Cardiovasc Surg. (2025) 169:1597–603. doi: 10.1016/j.jtcvs.2024.12.006 39675416

[5] Grosso F Cerbone L Curioni-Fontecedro A . MARS 2 trial: the future of pleurectomy decortication in pleural mesothelioma. Lancet Respir Med. (2024) 12:423–4. doi: 10.1016/S2213-2600(24)00146-2 38740043

[6] Baas P Scherpereel A Nowak AK Fujimoto N Peters S Tsao AS . First-line nivolumab plus ipilimumab in unresectable Malignant pleural mesothelioma (CheckMate 743): a multicentre, randomised, open-label, phase 3 trial. Lancet. (2021) 397:375–86. doi: 10.1016/S0140-6736(20)32714-8 33485464

[7] Chu Q Perrone F Greillier L Tu W Piccirillo MC Grosso F . Pembrolizumab plus chemotherapy versus chemotherapy in untreated advanced pleural mesothelioma in Canada, Italy, and France: a phase 3, open-label, randomised controlled trial. Lancet. (2023) 402:2295–306. doi: 10.1016/S0140-6736(23)01613-6 37931632

[8] Delsignore M Cassinari G Revello S Cerbone L Grosso F Arsura M . Determinants of response to immune checkpoint blockade in pleural mesothelioma: molecular, immunological, and clinical perspectives. Cancers (Basel). (2025) 17:4020. doi: 10.3390/cancers17244020 41463268 PMC12731717

[9] Nowak AK Robinson BWS Lake RA . Synergy between chemotherapy and immunotherapy in the treatment of established murine solid tumors. Cancer Res. (2003) 63:4490–6. 12907622

[10] Lesterhuis WJ Salmons J Nowak AK Rozali EN Khong A Dick IM . Synergistic effect of CTLA-4 blockade and cancer chemotherapy in the induction of anti-tumor immunity. PloS One. (2013) 8:e61895. doi: 10.1371/journal.pone.0061895 23626745 PMC3633941

[11] Pasello G Zago G Lunardi F Urso L Kern I Vlacic G . Malignant pleural mesothelioma immune microenvironment and checkpoint expression: correlation with clinical-pathological features and intratumor heterogeneity over time. Ann Oncol. (2018) 29:1258–65. doi: 10.1093/annonc/mdy086 29514216

[12] Principe N Aston WJ Hope DE Tilsed CM Fisher SA Boon L . Comprehensive testing of chemotherapy and immune checkpoint blockade in preclinical cancer models identifies additive combinations. Front Immunol. (2022) 13:872295. doi: 10.3389/fimmu.2022.872295 35634282 PMC9132586

[13] Scherpereel A Baas P Nowak AK Tsao AS Fujimoto N Peters S . Five-year clinical outcomes with nivolumab plus ipilimumab versus chemotherapy as first-line treatment for unresectable pleural mesothelioma in CheckMate 743. J Clin Oncol. (2026) 44:742–9. doi: 10.1200/JCO-25-01328 41734361 PMC13001902

[14] Fiorilla I Martinotti S Todesco AM Bonsignore G Cavaletto M Patrone M . Chronic inflammation, oxidative stress and metabolic plasticity: three players driving the pro-tumorigenic microenvironment in Malignant mesothelioma. Cells. (2023) 12:2048. doi: 10.3390/cells12162048 37626858 PMC10453755

[15] Sekido Y . Molecular pathogenesis of Malignant mesothelioma. Carcinogenesis. (2013) 34:1413–9. doi: 10.1093/carcin/bgt166 23677068

[16] Hiltbrunner S Mannarino L Kirschner MB Opitz I Rigutto A Laure A . Tumor immune microenvironment and genetic alterations in mesothelioma. Front Oncol. (2021) 11:660039. doi: 10.3389/fonc.2021.660039 34249695 PMC8261295

[17] Horio D Minami T Kitai H Ishigaki H Higashiguchi Y Kondo N . Tumor-associated macrophage-derived inflammatory cytokine enhances Malignant potential of Malignant pleural mesothelioma. Cancer Sci. (2020) 111:2895–906. doi: 10.1111/cas.14523 32530527 PMC7419052

[18] Wang Y Faux SP Hallden G Kirn DH Houghton CE Lemoine NR . Interleukin-1beta and tumour necrosis factor-alpha promote the transformation of human immortalised mesothelial cells by erionite. Int J Oncol. (2004) 25:173–8. doi: 10.3892/ijo.25.1.173 15202003

[19] Désage A-L Karpathiou G Peoc’h M Froudarakis ME . The immune microenvironment of Malignant pleural mesothelioma: a literature review. Cancers (Basel). (2021) 13:3205. doi: 10.3390/cancers13133205 34206956 PMC8269097

[20] Harber J Kamata T Pritchard C Fennell D . Matter of TIME: the tumor-immune microenvironment of mesothelioma and implications for checkpoint blockade efficacy. J Immunother Cancer. (2021) 9:e003032. doi: 10.1136/jitc-2021-003032 34518291 PMC8438820

[21] Minnema-Luiting J Vroman H Aerts J Cornelissen R . Heterogeneity in immune cell content in Malignant pleural mesothelioma. Int J Mol Sci. (2018) 19:1041. doi: 10.3390/ijms19041041 29601534 PMC5979422

[22] Santiago-Sánchez GS Fabian KP Hodge JW . A landscape of checkpoint blockade resistance in cancer: underlying mechanisms and current strategies to overcome resistance. Cancer Biol Ther. (2024) 25:2308097. doi: 10.1080/15384047.2024.2308097 38306161 PMC10841019

[23] Galon J Bruni D . Approaches to treat immune hot, altered and cold tumours with combination immunotherapies. Nat Rev Drug Discov. (2019) 18:197–218. doi: 10.1038/s41573-018-0007-y 30610226

[24] Napoli F Listì A Zambelli V Witel G Bironzo P Papotti M . Pathological characterization of tumor immune microenvironment (TIME) in Malignant pleural mesothelioma. Cancers (Basel). (2021) 13:2564. doi: 10.3390/cancers13112564 34073720 PMC8197227

[25] Salaroglio IC Kopecka J Napoli F Pradotto M Maletta F Costardi L . Potential diagnostic and prognostic role of microenvironment in Malignant pleural mesothelioma. J Thorac Oncol. (2019) 14:1458–71. doi: 10.1016/j.jtho.2019.03.029 31078776

[26] Waldman AD Fritz JM Lenardo MJ . A guide to cancer immunotherapy: from T cell basic science to clinical practice. Nat Rev Immunol. (2020) 20:651–68. doi: 10.1038/s41577-020-0306-5 32433532 PMC7238960

[27] Espinosa-Carrasco G Chiu E Scrivo A Zumbo P Dave A Betel D . Intratumoral immune triads are required for immunotherapy-mediated elimination of solid tumors. Cancer Cell. (2024) 42:1202–1216.e8. doi: 10.1016/j.ccell.2024.05.025 38906155 PMC11413804

[28] Yin Y Sakakibara R Honda T Kirimura S Daroonpan P Kobayashi M . High density and proximity of CD8+ T cells to tumor cells are correlated with better response to nivolumab treatment in metastatic pleural mesothelioma. Thorac Cancer. (2023) 14:1991–2000. doi: 10.1111/1759-7714.14981 37253418 PMC10344741

[29] Torricelli F Spada F Bishop C Todd K Nonaka D Petrov N . The phenogenomic landscapes of pleural mesothelioma tumor microenvironment predict clinical outcomes. J Transl Med. (2025) 23:208. doi: 10.1186/s12967-025-06193-z 39980060 PMC11844119

[30] Xu D Gao Y Yang H Spils M Marti TM Losmanová T . BAP1 deficiency inflames the tumor immune microenvironment and is a candidate biomarker for immunotherapy response in Malignant pleural mesothelioma. JTO Clin Res Rep. (2024) 5:100672. doi: 10.1016/j.jtocrr.2024.100672 38715965 PMC11070913

[31] Mansfield AS Brown RJ Sammon C Daumont MJ McKenna M Sanzari JK . The predictive and prognostic nature of programmed death-ligand 1 in Malignant pleural mesothelioma: a systematic literature review. JTO Clin Res Rep. (2022) 3:100315. doi: 10.1016/j.jtocrr.2022.100315 35516726 PMC9062484

[32] Alley EW Lopez J Santoro A Morosky A Saraf S Piperdi B . Clinical safety and activity of pembrolizumab in patients with Malignant pleural mesothelioma (KEYNOTE-028): preliminary results from a non-randomised, open-label, phase 1b trial. Lancet Oncol. (2017) 18:623–30. doi: 10.1016/S1470-2045(17)30169-9 28291584

[33] Hassan R Thomas A Nemunaitis JJ Patel MR Bennouna J Chen FL . Efficacy and safety of avelumab treatment in patients with advanced unresectable mesothelioma: phase 1b results from the JAVELIN solid tumor trial. JAMA Oncol. (2019) 5:351–7. doi: 10.1001/jamaoncol.2018.5428 30605211 PMC6439847

[34] Marcq E Siozopoulou V De Waele J van Audenaerde J Zwaenepoel K Santermans E . Prognostic and predictive aspects of the tumor immune microenvironment and immune checkpoints in Malignant pleural mesothelioma. Oncoimmunology. (2017) 6:e1261241. doi: 10.1080/2162402X.2016.1261241 28197385 PMC5283621

[35] Brcic L Klikovits T Megyesfalvi Z Mosleh B Sinn K Hritcu R . Prognostic impact of PD-1 and PD-L1 expression in Malignant pleural mesothelioma: an international multicenter study. Transl Lung Cancer Res. (2021) 10:1594–607. doi: 10.21037/tlcr-20-1114 34012777 PMC8107750

[36] Khanna S Thomas A Abate-Daga D Zhang J Morrow B Steinberg SM . Malignant mesothelioma effusions are infiltrated by CD3+ T cells highly expressing PD-L1 and the PD-L1+ tumor cells within these effusions are susceptible to ADCC by the anti-PD-L1 antibody avelumab. J Thorac Oncol. (2016) 11:1993–2005. doi: 10.1016/j.jtho.2016.07.033 27544053 PMC5075512

[37] Carbotti G Dozin B Martini S Giordano C Scordamaglia F Croce M . IL-27 mediates PD-L1 expression and release by human mesothelioma cells. Cancers (Basel). (2021) 13:4011. doi: 10.3390/cancers13164011 34439164 PMC8393193

[38] Carosio R Fontana V Mastracci L Ferro P Grillo F Banelli B . Characterization of soluble PD-L1 in pleural effusions of mesothelioma patients: potential implications in the immune response and prognosis. J Cancer Res Clin Oncol. (2021) 147:459–68. doi: 10.1007/s00432-020-03457-7 33216211 PMC11801835

[39] Kurosaki T Chamoto K Suzuki S Kanemura H Mitani S Tanaka K . The combination of soluble forms of PD-1 and PD-L1 as a predictive marker of PD-1 blockade in patients with advanced cancers: a multicenter retrospective study. Front Immunol. (2023) 14:1325462. doi: 10.3389/fimmu.2023.1325462 38149256 PMC10750355

[40] Chiarucci C Cannito S Daffinà MG Amato G Giacobini G Cutaia O . Circulating levels of PD-L1 in mesothelioma patients from the NIBIT-MESO-1 study: correlation with survival. Cancers (Basel). (2020) 12:361. doi: 10.3390/cancers12020361 32033266 PMC7072596

[41] Homicsko K Zygoura P Norkin M Tissot S Shakarishvili N Popat S . PD-1-expressing macrophages and CD8 T cells are independent predictors of clinical benefit from PD-1 inhibition in advanced mesothelioma. J Immunother Cancer. (2023) 11:e007585. doi: 10.1136/jitc-2023-007585 37880184 PMC10603330

[42] Okita R Mimura-Kimura Y Kawamoto N Yamamoto N Umeda M Okada M . Effects of tumor-infiltrating CD8+ T cells, PD1/PD-L1 axis, and expression patterns of HLA class I on the prognosis of patients with Malignant pleural mesothelioma who underwent extra-pleural pneumonectomy. Cancer Immunol Immunother. (2023) 72:865–79. doi: 10.1007/s00262-022-03292-4 36115921 PMC10991632

[43] Chee J Watson MW Chopra A Nguyen B Cook AM Creaney J . Tumour associated lymphocytes in the pleural effusions of patients with mesothelioma express high levels of inhibitory receptors. BMC Res Notes. (2018) 11:864. doi: 10.1186/s13104-018-3953-x 30518402 PMC6282254

[44] Flieswasser T Van den Eynde A Van Audenaerde J De Waele J Lardon F Riether C . The CD70-CD27 axis in oncology: the new kids on the block. J Exp Clin Cancer Res. (2022) 41:12. doi: 10.1186/s13046-021-02215-y 34991665 PMC8734249

[45] Hu J Li J Yang B Wang S Bao Y Yang Y . CD70: an emerging target for integrated cancer diagnosis and therapy. Clin Transl Med. (2025) 15:e70400. doi: 10.1002/ctm2.70400 40629902 PMC12238729

[46] Inaguma S Ueki A Lasota J Komura M Sheema AN Czapiewski P . CD70 and PD-L1 (CD274) co-expression predicts poor clinical outcomes in patients with pleural mesothelioma. J Pathol Clin Res. (2023) 9:195–207. doi: 10.1002/cjp2.310 36754859 PMC10073927

[47] Acharya N Sabatos-Peyton C Anderson AC . Tim-3 finds its place in the cancer immunotherapy landscape. J Immunother Cancer. (2020) 8:e000911. doi: 10.1136/jitc-2020-000911 32601081 PMC7326247

[48] Awad MM Jones RE Liu H Lizotte PH Ivanova EV Kulkarni M . Cytotoxic T cells in PD-L1-positive Malignant pleural mesotheliomas are counterbalanced by distinct immunosuppressive factors. Cancer Immunol Res. (2016) 4:1038–48. doi: 10.1158/2326-6066.CIR-16-0171 27856426

[49] Sobhani N Tardiel-Cyril DR Davtyan A Generali D Roudi R Li Y . CTLA-4 in regulatory T cells for cancer immunotherapy. Cancers (Basel). (2021) 13:1440. doi: 10.3390/cancers13061440 33809974 PMC8005092

[50] Calabrò L Morra A Fonsatti E Cutaia O Fazio C Annesi D . Efficacy and safety of an intensified schedule of tremelimumab for chemotherapy-resistant Malignant mesothelioma: an open-label, single-arm, phase 2 study. Lancet Respir Med. (2015) 3:301–9. doi: 10.1016/S2213-2600(15)00092-2 25819643

[51] Calabrò L Morra A Giannarelli D Amato G D’Incecco A Covre A . Tremelimumab combined with durvalumab in patients with mesothelioma (NIBIT-MESO-1): an open-label, non-randomised, phase 2 study. Lancet Respir Med. (2018) 6:451–60. doi: 10.1016/S2213-2600(18)30151-6 29773326

[52] Mankor JM Disselhorst MJ Poncin M Baas P Aerts JGJV Vroman H . Efficacy of nivolumab and ipilimumab in patients with Malignant pleural mesothelioma is related to a subtype of effector memory cytotoxic T cells: Translational evidence from two clinical trials. EBioMedicine. (2020) 62:103040. doi: 10.1016/j.ebiom.2020.103040 33166791 PMC7658658

[53] Peters S Scherpereel A Cornelissen R Oulkhouir Y Greillier L Kaplan MA . First-line nivolumab plus ipilimumab versus chemotherapy in patients with unresectable Malignant pleural mesothelioma: 3-year outcomes from CheckMate 743. Ann Oncol. (2022) 33:488–99. doi: 10.1016/j.annonc.2022.01.074 35124183

[54] Roncella S Laurent S Fontana V Ferro P Franceschini MC Salvi S . CTLA-4 in mesothelioma patients: tissue expression, body fluid levels and possible relevance as a prognostic factor. Cancer Immunol Immunother. (2016) 65:909–17. doi: 10.1007/s00262-016-1844-3 27207606 PMC11029598

[55] Checkpoint blockade regulates T cell fate by supporting co-stimulation. Nat Cancer. (2024) 5:1796–7. doi: 10.1038/s43018-024-00870-6 39681654

[56] Klampatsa A O’Brien SM Thompson JC Rao AS Stadanlick JE Martinez MC . Phenotypic and functional analysis of Malignant mesothelioma tumor-infiltrating lymphocytes. Oncoimmunology. (2019) 8:e1638211. doi: 10.1080/2162402X.2019.1638211 31428531 PMC6685523

[57] Corrigan DT Tanwar A Du M Martin AM Zang X . The B7-H3 (CD276) pathway: emerging biology and clinical therapeutics. Trends Pharmacol Sci. (2025) 46:975–88. doi: 10.1016/j.tips.2025.08.008 40946079 PMC12435905

[58] Aoki Y Arimura K Hiroshima K Sato Y Kondo M Tagaya E . CD276 as a critical independent biomarker and immune checkpoint inhibitor target in epithelioid mesothelioma-TCGA study. J Thorac Dis. (2025) 17:109–20. doi: 10.21037/jtd-24-1598 39975728 PMC11833596

[59] Ma X Lembersky D Kim ES Becich MJ Testa JR Bruno TC . Spatial landscape of Malignant pleural and peritoneal mesothelioma tumor immune microenvironments. Cancer Res Commun. (2024) 4:2133–46. doi: 10.1158/2767-9764.CRC-23-0524 38994676 PMC11328914

[60] Arimura K Hiroshima K Nagashima Y Nakazawa T Ogihara A Orimo M . LAG3 is an independent prognostic biomarker and potential target for immune checkpoint inhibitors in Malignant pleural mesothelioma: a retrospective study. BMC Cancer. (2023) 23:1206. doi: 10.1186/s12885-023-11636-1 38062416 PMC10704683

[61] Marcq E Waele JD Audenaerde JV Lion E Santermans E Hens N . Abundant expression of TIM-3, LAG-3, PD-1 and PD-L1 as immunotherapy checkpoint targets in effusions of mesothelioma patients. Oncotarget. (2017) 8:89722–35. doi: 10.18632/oncotarget.21113 29163783 PMC5685704

[62] Nowak EC Lines JL Varn FS Deng J Sarde A Mabaera R . Immunoregulatory functions of VISTA. Immunol Rev. (2017) 276:66–79. doi: 10.1111/imr.12525 28258694 PMC5702497

[63] Zhang RJ Kim TK . VISTA-mediated immune evasion in cancer. Exp Mol Med. (2024) 56:2348–56. doi: 10.1038/s12276-024-01336-6 39482534 PMC11612309

[64] Hmeljak J Sanchez-Vega F Hoadley KA Shih J Stewart C Heiman D . Integrative molecular characterization of Malignant pleural mesothelioma. Cancer Discov. (2018) 8:1548–65. doi: 10.1158/2159-8290.CD-18-0804 30322867 PMC6310008

[65] Muller S Victoria Lai W Adusumilli PS Desmeules P Frosina D Jungbluth A . V-domain Ig-containing suppressor of T-cell activation (VISTA), a potentially targetable immune checkpoint molecule, is highly expressed in epithelioid Malignant pleural mesothelioma. Mod Pathol. (2020) 33:303–11. doi: 10.1038/s41379-019-0364-z 31537897 PMC8366498

[66] Osmanbeyoglu HU Palmer D Sagan A Sementino E Becich MJ Testa JR . Isolated BAP1 genomic alteration in Malignant pleural mesothelioma predicts distinct immunogenicity with implications for immunotherapeutic response. Cancers (Basel). (2022) 14:5626. doi: 10.3390/cancers14225626 36428720 PMC9688367

[67] Kamiya T Seow SV Wong D Robinson M Campana D . Blocking expression of inhibitory receptor NKG2A overcomes tumor resistance to NK cells. J Clin Invest. (2019) 129:2094–106. doi: 10.1172/JCI123955 30860984 PMC6486333

[68] Giotti B Dolasia K Zhao W Cai P Sweeney R Merritt E . Single-cell view of tumor microenvironment gradients in pleural mesothelioma. Cancer Discov. (2024) 14:2262–78. doi: 10.1158/2159-8290.CD-23-0017 38959428 PMC13109001

[69] Galluzzi L Smith KN Liston A Garg AD . The diversity of CD8+ T cell dysfunction in cancer and viral infection. Nat Rev Immunol. (2025) 25:662–79. doi: 10.1038/s41577-025-01161-6 40216888

[70] Klotz LV Weigert A Eichhorn F Allgäuer M Muley T Shah R . Impact of T cell ratios on survival in pleural mesothelioma: insights from tumor microenvironment analysis. Cancers (Basel). (2024) 16:3418. doi: 10.3390/cancers16193418 39410037 PMC11476058

[71] Noordam L Kaijen MEH Bezemer K Cornelissen R Maat LAPWM Hoogsteden HC . Low-dose cyclophosphamide depletes circulating naïve and activated regulatory T cells in Malignant pleural mesothelioma patients synergistically treated with dendritic cell-based immunotherapy. Oncoimmunology. (2018) 7:e1474318. doi: 10.1080/2162402X.2018.1474318 30524884 PMC6279421

[72] Kohno M Murakami J Wu L Chan M-L Yun Z Cho BCJ . Foxp3+ regulatory T cell depletion after nonablative oligofractionated irradiation boosts the abscopal effects in murine Malignant mesothelioma. J Immunol. (2020) 205:2519–31. doi: 10.4049/jimmunol.2000487 32948683

[73] Wu L Wu MO De la Maza L Yun Z Yu J Zhao Y . Targeting the inhibitory receptor CTLA-4 on T cells increased abscopal effects in murine mesothelioma model. Oncotarget. (2015) 6:12468–80. doi: 10.18632/oncotarget.3487 25980578 PMC4494951

[74] Anraku M Tagawa T Wu L Yun Z Keshavjee S Zhang L . Synergistic antitumor effects of regulatory T cell blockade combined with pemetrexed in murine Malignant mesothelioma. J Immunol. (2010) 185:956–66. doi: 10.4049/jimmunol.0900437 20548032

[75] Lu J Luo Y Rao D Wang T Lei Z Chen X . Myeloid-derived suppressor cells in cancer: therapeutic targets to overcome tumor immune evasion. Exp Hematol Oncol. (2024) 13:39. doi: 10.1186/s40164-024-00505-7 38609997 PMC11010322

[76] Veltman JD Lambers MEH van Nimwegen M Hendriks RW Hoogsteden HC Aerts JGJV . COX-2 inhibition improves immunotherapy and is associated with decreased numbers of myeloid-derived suppressor cells in mesothelioma. Celecoxib influences MDSC function. BMC Cancer. (2010) 10:464. doi: 10.1186/1471-2407-10-464 20804550 PMC2939552

[77] Dhupar R Okusanya OT Eisenberg SH Monaco SE Ruffin AT Liu D . Characteristics of Malignant pleural effusion resident CD8+ T cells from a heterogeneous collection of tumors. Int J Mol Sci. (2020) 21:6178. doi: 10.3390/ijms21176178 32867034 PMC7503595

[78] de Gooijer CJ van der Noort V Stigt JA Baas P Biesma B Cornelissen R . Switch-maintenance gemcitabine after first-line chemotherapy in patients with Malignant mesothelioma (NVALT19): an investigator-initiated, randomised, open-label, phase 2 trial. Lancet Respir Med. (2021) 9:585–92. doi: 10.1016/S2213-2600(20)30362-3 33515500

[79] Dammeijer F De Gooijer CJ van Gulijk M Lukkes M Klaase L Lievense LA . Immune monitoring in mesothelioma patients identifies novel immune-modulatory functions of gemcitabine associating with clinical response. EBioMedicine. (2021) 64:103160. doi: 10.1016/j.ebiom.2020.103160 33516644 PMC7910686

[80] Hashemi Goradel N Najafi M Salehi E Farhood B Mortezaee K . Cyclooxygenase-2 in cancer: a review. J Cell Physiol. (2019) 234:5683–99. doi: 10.1002/jcp.27411 30341914

[81] Marrogi A Pass HI Khan M Metheny-Barlow LJ Harris CC Gerwin BI . Human mesothelioma samples overexpress both cyclooxygenase-2 (COX-2) and inducible nitric oxide synthase (NOS2): *in vitro* antiproliferative effects of a COX-2 inhibitor. Cancer Res. (2000) 60:3696–700. 10919635

[82] Obermajer N Kalinski P . Key role of the positive feedback between PGE(2) and COX2 in the biology of myeloid-derived suppressor cells. Oncoimmunology. (2012) 1:762–4. doi: 10.4161/onci.19681 22934275 PMC3429587

[83] Tan Z Liu L Chiu MS Cheung K-W Yan CW Yu Z . Virotherapy-recruited PMN-MDSC infiltration of mesothelioma blocks antitumor CTL by IL-10-mediated dendritic cell suppression. Oncoimmunology. (2019) 8:e1518672. doi: 10.1080/2162402X.2018.1518672 30546960 PMC6287797

[84] Tedesco J Jaradeh M Vigneswaran WT . Malignant pleural mesothelioma: current understanding of the immune microenvironment and treatments of a rare disease. Cancers (Basel). (2022) 14:4415. doi: 10.3390/cancers14184415 36139575 PMC9496741

[85] Wu L Kohno M Murakami J Zia A Allen J Yun H . Defining and targeting tumor-associated macrophages in Malignant mesothelioma. Proc Natl Acad Sci USA. (2023) 120:e2210836120. doi: 10.1073/pnas.2210836120 36821580 PMC9992826

[86] Sheng R Yin Y Wang X . Mesothelial and immune cells interplay in the tumor microenvironment. Trends Mol Med. (2025) 31:895–908. doi: 10.1016/j.molmed.2025.03.014 40307075

[87] Porta C Jean D Blanquart C . Editorial: Focus on Malignant pleural mesothelioma immunology and immunotherapy. Front Immunol. (2023) 14:1251384. doi: 10.3389/fimmu.2023.1251384 37554331 PMC10406124

[88] Laberiano-Fernandez C Baldavira CM MaChado-Rugolo J Tamegnon A Pandurengan RK Ab’Saber AM . The immunological landscape of M1 and M2 macrophages and their spatial distribution in patients with Malignant pleural mesothelioma. Cancers (Basel). (2023) 15:5116. doi: 10.3390/cancers15215116 37958292 PMC10650059

[89] Cersosimo F Barbarino M Lonardi S Vermi W Giordano A Bellan C . Mesothelioma Malignancy and the microenvironment: molecular mechanisms. Cancers (Basel). (2021) 13:5664. doi: 10.3390/cancers13225664 34830817 PMC8616064

[90] Calabrò L Bronte G Grosso F Cerbone L Delmonte A Nicolini F . Immunotherapy of mesothelioma: the evolving change of a long-standing therapeutic dream. Front Immunol. (2023) 14:1333661. doi: 10.3389/fimmu.2023.1333661 38259475 PMC10800748

[91] Nowak AK Brosseau S Cook A Zalcman G . Antiangiogeneic strategies in mesothelioma. Front Oncol. (2020) 10:126. doi: 10.3389/fonc.2020.00126 32133285 PMC7040194

[92] Magkouta S Kollintza A Moschos C Spella M Skianis I Pappas A . Role of angiopoietins in mesothelioma progression. Cytokine. (2019) 118:99–106. doi: 10.1016/j.cyto.2018.08.006 30201261

[93] Kumar-Singh S Weyler J Martin MJ Vermeulen PB Van Marck E . Angiogenic cytokines in mesothelioma: a study of VEGF, FGF-1 and -2, and TGF beta expression. J Pathol. (1999) 189:72–8. doi: 10.1002/(SICI)1096-9896(199909)189:1<72::AID-PATH401>3.0.CO;2-0 10451491

[94] Melincovici CS Boşca AB Şuşman S Mărginean M Mihu C Istrate M . Vascular endothelial growth factor (VEGF) - key factor in normal and pathological angiogenesis. Rom J Morphol Embryol. (2018) 59:455–67. 30173249

[95] Vimalraj S . A concise review of VEGF, PDGF, FGF, Notch, angiopoietin, and HGF signalling in tumor angiogenesis with a focus on alternative approaches and future directions. Int J Biol Macromol. (2022) 221:1428–38. doi: 10.1016/j.ijbiomac.2022.09.129 36122781

[96] Strizzi L Catalano A Vianale G Orecchia S Casalini A Tassi G . Vascular endothelial growth factor is an autocrine growth factor in human Malignant mesothelioma. J Pathol. (2001) 193:468–75. doi: 10.1002/path.824 11276005

[97] Lertkiatmongkol P Liao D Mei H Hu Y Newman PJ . Endothelial functions of platelet/endothelial cell adhesion molecule-1 (CD31). Curr Opin Hematol. (2016) 23:253–9. doi: 10.1097/MOH.0000000000000239 27055047 PMC4986701

[98] Gayen S . Malignant pleural effusion: presentation, diagnosis, and management. Am J Med. (2022) 135:1188–92. doi: 10.1016/j.amjmed.2022.04.017 35576996

[99] Mathilakathu A Wessolly M Mairinger E Uebner H Kreidt D Brcic L . Cancer-associated fibroblasts regulate kinase activity in mesothelioma cell lines via paracrine signaling and thereby dictate cell faith and behavior. Int J Mol Sci. (2022) 23:3278. doi: 10.3390/ijms23063278 35328699 PMC8949651

[100] Jakab M Augustin HG . Understanding angiodiversity: insights from single cell biology. Development. (2020) 147:dev146621. doi: 10.1242/dev.146621 32792338

[101] Arnould M Neilly MDJ Blyth KG Jean D . Intratumour, anatomical and temporal heterogeneity in mesothelioma. Thorax. (2025):thorax-2024-222052. doi: 10.1136/thorax-2024-222052 41176322

[102] Oehl K Vrugt B Opitz I Meerang M . Heterogeneity in Malignant pleural mesothelioma. Int J Mol Sci. (2018) 19:1603. doi: 10.3390/ijms19061603 29848954 PMC6032160

[103] Meiller C Montagne F Hirsch TZ Caruso S de Wolf J Bayard Q . Multi-site tumor sampling highlights molecular intra-tumor heterogeneity in Malignant pleural mesothelioma. Genome Med. (2021) 13:113. doi: 10.1186/s13073-021-00931-w 34261524 PMC8281651

[104] Li J He H Zhang Y Azhar MS Wang Y . Comprehensive analysis reveals prognostic gene set that could impacts the response to chemotherapy and immunotherapy outcomes in Malignant pleural mesothelioma. Clin Exp Med. (2025) 26:22. doi: 10.1007/s10238-025-01949-9 41249691 PMC12628432

[105] Zhang M Luo J-L Sun Q Harber J Dawson AG Nakas A . Author correction: Clonal architecture in mesothelioma is prognostic and shapes the tumour microenvironment. Nat Commun. (2021) 12:3569. doi: 10.1038/s41467-021-23867-6 34099722 PMC8185116

[106] Blum Y Meiller C Quetel L Elarouci N Ayadi M Tashtanbaeva D . Dissecting heterogeneity in Malignant pleural mesothelioma through histo-molecular gradients for clinical applications. Nat Commun. (2019) 10:1333. doi: 10.1038/s41467-019-09307-6 30902996 PMC6430832

[107] Kiyotani K Park J-H Inoue H Husain A Olugbile S Zewde M . Integrated analysis of somatic mutations and immune microenvironment in Malignant pleural mesothelioma. Oncoimmunology. (2017) 6:e1278330. doi: 10.1080/2162402X.2016.1278330 28344893 PMC5353938

[108] Bueno R Stawiski EW Goldstein LD Durinck S De Rienzo A Modrusan Z . Comprehensive genomic analysis of Malignant pleural mesothelioma identifies recurrent mutations, gene fusions and splicing alterations. Nat Genet. (2016) 48:407–16. doi: 10.1038/ng.3520 26928227

[109] Fennell DA Hill K Zhang M Poile C Ewings S Baitei EY . Constitutive inflammation and epithelial-mesenchymal transition dictate sensitivity to nivolumab in CONFIRM: a placebo-controlled, randomised phase III trial. Nat Commun. (2025) 16:6688. doi: 10.1038/s41467-025-61691-4 40691143 PMC12280041

[110] Dagogo-Jack I Mitchell O Codd E Li A Mitchell D Flynn SE . Immune composition and immunotherapy outcomes of mesothelioma with BAP1, CDKN2A, MTAP, and NF2 alterations. J Thorac Oncol. (2025) 20:1615–25. doi: 10.1016/j.jtho.2025.06.018 40578509

[111] Genugten J Faulkner D Hahne JC Poile C Wessels L Fennell DA . Discovery of actionable drug targets to enhance T-cell infiltration and immune checkpoint blockade efficacy in pleural mesothelioma. Lung Cancer. (2025) 209:108769. doi: 10.1016/j.lungcan.2025.108769 41043322

[112] Genova C Dellepiane C Carrega P Sommariva S Ferlazzo G Pronzato P . Therapeutic implications of tumor microenvironment in lung cancer: Focus on immune checkpoint blockade. Front Immunol. (2021) 12:799455. doi: 10.3389/fimmu.2021.799455 35069581 PMC8777268

[113] Yang K Yang T Yang T Yuan Y Li F . Unraveling tumor microenvironment heterogeneity in Malignant pleural mesothelioma identifies biologically distinct immune subtypes enabling prognosis determination. Front Oncol. (2022) 12:995651. doi: 10.3389/fonc.2022.995651 36237331 PMC9552848

[114] Alay A Cordero D Hijazo-Pechero S Aliagas E Lopez-Doriga A Marín R . Integrative transcriptome analysis of Malignant pleural mesothelioma reveals a clinically relevant immune-based classification. J Immunother Cancer. (2021) 9:e001601. doi: 10.1136/jitc-2020-001601 33632900 PMC7908918

[115] Behrouzfar K Mutsaers SE Chin WL Patrick K Ng IT Pixley FJ . Mesothelioma survival prediction based on a six-gene transcriptomic signature. iScience. (2024) 27:111011. doi: 10.1016/j.isci.2024.111011 39474071 PMC11519557

[116] Severson DT Freyaldenhoven S Wadowski B Hung YP Hughes T Yeap BY . Multiomic, histologic, and scRNA-seq profiling of pleural mesothelioma reveals negative prognosis associated with a novel uncommitted molecular phenotype. J Thorac Oncol. (2025):S1556-0864(25)02937–5. doi: 10.1016/j.jtho.2025.11.019 41319863 PMC12853269

[117] Ozirmak Lermi N Molina Ayala M Hernandez S Lu W Khan K Serrano A . Comparison of imaging based single-cell resolution spatial transcriptomics profiling platforms using formalin-fixed paraffin-embedded tumor samples. Nat Commun. (2025) 16:8499. doi: 10.1038/s41467-025-63414-1 41006245 PMC12474935

[118] Torricelli F Donati B Manicardi V Gugnoni M Reggiani F Manzotti G . CDKN2A deletion is associated with immune desertification in diffuse pleural mesothelioma. J Exp Clin Cancer Res. (2025) 44:256. doi: 10.1186/s13046-025-03522-4 40877968 PMC12392524

[119] Torricelli F Donati B Reggiani F Manicardi V Piana S Valli R . Spatially resolved, high-dimensional transcriptomics sorts out the evolution of biphasic Malignant pleural mesothelioma: new paradigms for immunotherapy. Mol Cancer. (2023) 22:114. doi: 10.1186/s12943-023-01816-9 37460925 PMC10351128

[120] Shek D Gloss B Lai J Ma L Zhang HE Carlino MS . Identification and characterisation of infiltrating immune cells in Malignant pleural mesothelioma using spatial transcriptomics. Methods Protoc. (2023) 6:35. doi: 10.3390/mps6020035 37104017 PMC10146955

[121] Patel AP Tirosh I Trombetta JJ Shalek AK Gillespie SM Wakimoto H . Single-cell RNA-seq highlights intratumoral heterogeneity in primary glioblastoma. Science. (2014) 344:1396–401. doi: 10.1126/science.1254257 24925914 PMC4123637

[122] Ma J Zhang H Yu L Guo Y Ming J Guo Z . Multi-omics integration reveals CYTL1 and H6PD as key regulators of tumor metabolism in mesothelioma. Genes Genomics. (2025) 47:1049–63. doi: 10.1007/s13258-025-01667-2 40853611

[123] Alay A Marín R Aliagas E Gausachs M Ruiz-Gil M Macia I . Single-cell RNA-sequencing as a potential approach for studying intratumor heterogeneity in pleural mesothelioma. Lung Cancer. (2025) 207:108679. doi: 10.1016/j.lungcan.2025.108679 40782703

[124] Liu J Liu Y Lu Y Zhang W Yan J Lu B . A novel mesothelioma molecular classification based on Malignant cell differentiation. Cancer Cell Int. (2025) 25:235. doi: 10.1186/s12935-025-03816-9 40555979 PMC12188665

[125] Zhang H Yu L Guo Y Ming J Guo Z . DNA methylation-regulated HLA-C expression modulates immune responses and metabolic alterations to influence prognosis in mesothelioma. Cancer Immunol Immunother. (2025) 74:158. doi: 10.1007/s00262-025-04012-4 40131544 PMC11937463

[126] Chin WL Cook AM Chee J Principe N Hoang TS Kidman J . Coupling of response biomarkers between tumor and peripheral blood in patients undergoing chemoimmunotherapy. Cell Rep Med. (2025) 6:101882. doi: 10.1016/j.xcrm.2024.101882 39731918 PMC11866441

[127] Obacz J Valer JA Nibhani R Adams TS Schupp JC Veale N . Single-cell transcriptomic analysis of human pleura reveals stromal heterogeneity and informs *in vitro* models of mesothelioma. Eur Respir J. (2024) 63:2300143. doi: 10.1183/13993003.00143-2023 38212075 PMC10809128

[128] Kao SC Cheng YY Williams M Kirschner MB Madore J Lum T . Tumor suppressor microRNAs contribute to the regulation of PD-L1 expression in Malignant pleural mesothelioma. J Thorac Oncol. (2017) 12:1421–33. doi: 10.1016/j.jtho.2017.05.024 28629895

[129] Costa C Indovina P Mattioli E Forte IM Iannuzzi CA Luzzi L . P53-regulated miR-320a targets PDL1 and is downregulated in Malignant mesothelioma. Cell Death Dis. (2020) 11:748. doi: 10.1038/s41419-020-02940-w 32929059 PMC7490273

[130] Truini A Coco S Nadal E Genova C Mora M Dal Bello MG . Downregulation of miR-99a/let-7c/miR-125b miRNA cluster predicts clinical outcome in patients with unresected Malignant pleural mesothelioma. Oncotarget. (2017) 8:68627–40. doi: 10.18632/oncotarget.19800 28978143 PMC5620283

[131] Ramírez-Salazar EG Salinas-Silva LC Vázquez-Manríquez ME Gayosso-Gómez LV Negrete-Garcia MC Ramírez-Rodriguez SL . Analysis of microRNA expression signatures in Malignant pleural mesothelioma, pleural inflammation, and atypical mesothelial hyperplasia reveals common predictive tumorigenesis-related targets. Exp Mol Pathol. (2014) 97:375–85. doi: 10.1016/j.yexmp.2014.09.016 25236577

[132] Yang X Lin Y Dong B Li B Liu R Wang X . Establishment and bioinformatics analysis of a four-miRNA prognostic signature for pleural mesothelioma. J Cancer. (2024) 15:6505–20. doi: 10.7150/jca.101914 39668817 PMC11632982

[133] Ho K-H Huang T-W Shih C-M Lee Y-T Liu A-J Chen P-H . Glycolysis-associated lncRNAs identify a subgroup of cancer patients with poor prognoses and a high-infiltration immune microenvironment. BMC Med. (2021) 19:59. doi: 10.1186/s12916-021-01925-6 33627136 PMC7905662

[134] Duan W Wang K Duan Y Chen X Chu X Hu P . Combined analysis of RNA sequence and microarray data reveals a competing endogenous RNA network as novel prognostic markers in Malignant pleural mesothelioma. Front Oncol. (2021) 11:615234. doi: 10.3389/fonc.2021.615234 33968720 PMC8104912

[135] Yuan D Wei Z Wang Y Cheng F Zeng Y Yang L . DNA methylation regulator-meditated modification patterns define the distinct tumor microenvironment in lung adenocarcinoma. Front Oncol. (2021) 11:734873. doi: 10.3389/fonc.2021.734873 34552879 PMC8450540

[136] Ressler JM Tomasich E Hatziioannou T Ringl H Heller G Silmbrod R . DNA methylation signatures correlate with response to immune checkpoint inhibitors in metastatic melanoma. Target Oncol. (2024) 19:263–75. doi: 10.1007/s11523-024-01041-4 38401029 PMC10963567

[137] Lofiego MF Cannito S Fazio C Piazzini F Cutaia O Solmonese L . Epigenetic immune remodeling of mesothelioma cells: A new strategy to improve the efficacy of immunotherapy. Epigenomes. (2021) 5:27. doi: 10.3390/epigenomes5040027 34968251 PMC8715476

[138] Lofiego MF Tufano R Bello E Solmonese L Marzani F Piazzini F . DNA methylation status classifies pleural mesothelioma cells according to their immune profile: implication for precision epigenetic therapy. J Exp Clin Cancer Res. (2025) 44:58. doi: 10.1186/s13046-025-03310-0 39966970 PMC11834541

[139] Calabrò L Caruso FP Covre A Noviello TMR Lofiego MF Tufano R . Tumor DNA methylation subtypes predict immunotherapy outcomes in pleural mesothelioma patients in the NIBIT-EPI-MESO study. Nat Genet. (2026) 58:1100–11. doi: 10.1038/s41588-026-02580-4 42045690

[140] Panou V Gadiraju M Wolin A Weipert CM Skarda E Husain AN . Frequency of germline mutations in cancer susceptibility genes in Malignant mesothelioma. J Clin Oncol. (2018) 36:2863–71. doi: 10.1200/JCO.2018.78.5204 30113886 PMC6804864

[141] Carbone M Harbour JW Brugarolas J Bononi A Pagano I Dey A . Biological mechanisms and clinical significance of BAP1 mutations in human cancer. Cancer Discov. (2020) 10:1103–20. doi: 10.1158/2159-8290.CD-19-1220 32690542 PMC8006752

[142] Louie BH Kurzrock R . BAP1: Not just a BRCA1-associated protein. Cancer Treat Rev. (2020) 90:102091. doi: 10.1016/j.ctrv.2020.102091 32877777 PMC7655689

[143] Shrestha R Nabavi N Lin Y-Y Mo F Anderson S Volik S . BAP1 haploinsufficiency predicts a distinct immunogenic class of Malignant peritoneal mesothelioma. Genome Med. (2019) 11:8. doi: 10.1186/s13073-019-0620-3 30777124 PMC6378747

[144] Zauderer MG Jayakumaran G DuBoff M Zhang L Francis JH Abramson DH . Prevalence and preliminary validation of screening criteria to identify carriers of germline BAP1 mutations. J Thorac Oncol. (2019) 14:1989–94. doi: 10.1016/j.jtho.2019.07.002 31323388 PMC6823112

[145] Rusch A Ziltener G Nackaerts K Weder W Stahel RA Felley-Bosco E . Prevalence of BRCA-1 associated protein 1 germline mutation in sporadic Malignant pleural mesothelioma cases. Lung Cancer. (2015) 87:77–9. doi: 10.1016/j.lungcan.2014.10.017 25468148

[146] Orozco-Castaño C Mejía-Garcia A Tsao HM Bonilla DA Carvajal-Fierro C Bruges-Maya R . Transcriptional landscape of pleural mesothelioma patients in relation to NF2 gene mutational status. J Egypt Natl Canc Inst. (2025) 37:25. doi: 10.1186/s43046-025-00284-0 40484892 PMC13313441

[147] Papaetis GS Syrigos KN . Sunitinib: a multitargeted receptor tyrosine kinase inhibitor in the era of molecular cancer therapies. BioDrugs. (2009) 23:377–89. doi: 10.2165/11318860-000000000-00000 19894779

[148] Laurie SA Gupta A Chu Q Lee CW Morzycki W Feld R . Brief report: a phase II study of sunitinib in Malignant pleural mesothelioma. the NCIC Clinical Trials Group. J Thorac Oncol. (2011) 6:1950–4. doi: 10.1097/JTO.0b013e3182333df5 22005473

[149] Laurie SA Hao D Leighl NB Goffin J Khomani A Gupta A . A phase II trial of dovitinib in previously-treated advanced pleural mesothelioma: The Ontario Clinical Oncology Group. Lung Cancer. (2017) 104:65–9. doi: 10.1016/j.lungcan.2016.12.004 28213002

[150] Parikh K Mandrekar SJ Allen-Ziegler K Esplin B Tan AD Marchello B . A phase II study of pazopanib in patients with Malignant pleural mesothelioma: NCCTG N0623 (Alliance). Oncologist. (2020) 25:523–31. doi: 10.1634/theoncologist.2019-0574 31872928 PMC7288653

[151] Garland LL Chansky K Wozniak AJ Tsao AS Gadgeel SM Verschraegen CF . Phase II study of cediranib in patients with Malignant pleural mesothelioma: SWOG S0509. J Thorac Oncol. (2011) 6:1938–45. doi: 10.1097/JTO.0b013e318229586e 21964533 PMC3477852

[152] Campbell NP Kunnavakkam R Leighl N Vincent MD Gandara DR Koczywas M . Cediranib in patients with Malignant mesothelioma: a phase II trial of the University of Chicago Phase II Consortium. Lung Cancer. (2012) 78:76–80. doi: 10.1016/j.lungcan.2012.06.011 22831987 PMC4319647

[153] Tsao AS Miao J Wistuba II Vogelzang NJ Heymach JV Fossella FV . Phase II trial of cediranib in combination with cisplatin and pemetrexed in chemotherapy-naïve patients with unresectable Malignant pleural mesothelioma (SWOG S0905). J Clin Oncol. (2019) 37:2537–47. doi: 10.1200/JCO.19.00269 31386610 PMC7001793

[154] Grosso F Steele N Novello S Nowak AK Popat S Greillier L . Nintedanib plus pemetrexed/cisplatin in patients with Malignant pleural mesothelioma: Phase II results from the randomized, placebo-controlled LUME-Meso trial. J Clin Oncol. (2017) 35:3591–600. doi: 10.1200/JCO.2017.72.9012 28892431

[155] Scagliotti GV Gaafar R Nowak AK Nakano T van Meerbeeck J Popat S . Nintedanib in combination with pemetrexed and cisplatin for chemotherapy-naive patients with advanced Malignant pleural mesothelioma (LUME-Meso): a double-blind, randomised, placebo-controlled phase 3 trial. Lancet Respir Med. (2019) 7:569–80. doi: 10.1016/S2213-2600(19)30139-0 31103412

[156] Wozniak AJ Schneider B Kalemkerian GP Daly B Chen W Ventimiglia J . Short report of a phase II trial of nintedanib in recurrent Malignant pleural mesothelioma (MPM). Clin Lung Cancer. (2023) 24:563–7. doi: 10.1016/j.cllc.2023.04.004 37301693 PMC11233026

[157] Zalcman G Mazieres J Margery J Greillier L Audigier-Valette C Moro-Sibilot D . Bevacizumab for newly diagnosed pleural mesothelioma in the Mesothelioma Avastin Cisplatin Pemetrexed Study (MAPS): a randomised, controlled, open-label, phase 3 trial. Lancet. (2016) 387:1405–14. doi: 10.1016/S0140-6736(15)01238-6 26719230

[158] Pinto C Zucali PA Pagano M Grosso F Pasello G Garassino MC . Gemcitabine with or without ramucirumab as second-line treatment for Malignant pleural mesothelioma (RAMES): a randomised, double-blind, placebo-controlled, phase 2 trial. Lancet Oncol. (2021) 22:1438–47. doi: 10.1016/S1470-2045(21)00404-6 34499874

[159] Danlos F-X Texier M Job B Mouraud S Cassard L Baldini C . Genomic instability and protumoral inflammation are associated with primary resistance to anti-PD-1 + antiangiogenesis in Malignant pleural mesothelioma. Cancer Discov. (2023) 13:858–79. doi: 10.1158/2159-8290.CD-22-0886 36669143 PMC10068454

[160] Douma L-A Lalezari F van der Noort V de Vries JF Monkhorst K Smesseim I . Pembrolizumab plus lenvatinib in second-line and third-line patients with pleural mesothelioma (PEMMELA): a single-arm phase 2 study. Lancet Oncol. (2023) 24:1219–28. doi: 10.1016/S1470-2045(23)00446-1 37844598

[161] Felip E Popat S Dafni U Ribi K Pope A Cedres S . A randomised phase III study of bevacizumab and carboplatin-pemetrexed chemotherapy with or without atezolizumab as first-line treatment for advanced pleural mesothelioma: results of the ETOP 13-18 BEAT-meso trial. Ann Oncol. (2025) 36:548–60. doi: 10.1016/j.annonc.2024.12.014 39814199

[162] Dudek AZ Xi MX Scilla KA Mamdani H Creelan BC Saltos A . Phase 2 trial of nivolumab and ramucirumab for relapsed mesothelioma: HCRN-LUN15-299. JTO Clin Res Rep. (2023) 4:100584. doi: 10.1016/j.jtocrr.2023.100584 38046376 PMC10689266

[163] Takagawa T Takahashi K Orimoto Y Morisaki Y Suna S Daimon T . Pembrolizumab plus platinum doublet chemotherapy with lenvatinib in unresectable pleural mesothelioma as first-line treatment (PENINSULA): a study protocol for a single-arm, multi-institutional, phase II clinical trial. Clin Lung Cancer. (2026) 27:75–9. doi: 10.1016/j.cllc.2025.12.010 41604907

[164] Labrijn AF Janmaat ML Reichert JM Parren PWHI . Bispecific antibodies: a mechanistic review of the pipeline. Nat Rev Drug Discov. (2019) 18:585–608. doi: 10.1038/s41573-019-0028-1 31175342

[165] Klein C Brinkmann U Reichert JM Kontermann RE . The present and future of bispecific antibodies for cancer therapy. Nat Rev Drug Discov. (2024) 23:301–19. doi: 10.1038/s41573-024-00896-6 38448606

[166] Zhong T Zhang L Huang Z Pang X Jin C Liu W . Design of a fragment crystallizable-engineered tetravalent bispecific antibody targeting programmed cell death-1 and vascular endothelial growth factor with cooperative biological effects. iScience. (2025) 28:111722. doi: 10.1016/j.isci.2024.111722 40034861 PMC11872405

[167] Monnet I Breau J-L Moro D Lena H Eymard J-C Ménard O . Intrapleural infusion of activated macrophages and gamma-interferon in Malignant pleural mesothelioma: a phase II study. Chest. (2002) 121:1921–7. doi: 10.1378/chest.121.6.1921 12065358

[168] Ponce S Cedrés S Ricordel C Isambert N Viteri S Herrera-Juarez M . ONCOS-102 plus pemetrexed and platinum chemotherapy in Malignant pleural mesothelioma: a randomized phase 2 study investigating clinical outcomes and the tumor microenvironment. J Immunother Cancer. (2023) 11:e007552. doi: 10.1136/jitc-2023-007552 37661097 PMC10476122

[169] Luke JJ Barlesi F Chung K Tolcher AW Kelly K Hollebecque A . Phase I study of ABBV-428, a mesothelin-CD40 bispecific, in patients with advanced solid tumors. J Immunother Cancer. (2021) 9:e002015. doi: 10.1136/jitc-2020-002015 33608377 PMC7898862

[170] Aerts JG Belderbos R Baas P Scherpereel A Bezemer K Enninga I . Dendritic cells loaded with allogeneic tumour cell lysate plus best supportive care versus best supportive care alone in patients with pleural mesothelioma as maintenance therapy after chemotherapy (DENIM): a multicentre, open-label, randomised, phase 2/3 study. Lancet Oncol. (2024) 25:865–78. doi: 10.1016/S1470-2045(24)00191-8 38848742

[171] Van den Bossche J De Laere M Deschepper K Germonpré P Valcke Y Lamont J . Integration of the PD-L1 inhibitor atezolizumab and WT1/DC vaccination into standard-of-care first-line treatment for patients with epithelioid Malignant pleural mesothelioma-protocol of the Immuno-MESODEC study. PloS One. (2024) 19:e0307204. doi: 10.1371/journal.pone.0307204 39008481 PMC11249236

[172] Schöffski P Tan DSW Martín M Ochoa-de-Olza M Sarantopoulos J Carvajal RD . Phase I/II study of the LAG-3 inhibitor ieramilimab (LAG525) ± anti-PD-1 spartalizumab (PDR001) in patients with advanced Malignancies. J Immunother Cancer. (2022) 10:e003776. doi: 10.1136/jitc-2021-003776 35217575 PMC8883259

[173] Lin C-C Garralda E Schöffski P Hong DS Siu LL Martin M . A phase 2, multicenter, open-label study of anti-LAG-3 ieramilimab in combination with anti-PD-1 spartalizumab in patients with advanced solid Malignancies. Oncoimmunology. (2024) 13:2290787. doi: 10.1080/2162402X.2023.2290787 38170160 PMC10761073

[174] Shi H Yu T-K Johnson B Selvamani SP Zhuang L Lee K . A combination of PD-1 and TIGIT immune checkpoint inhibitors elicits a strong anti-tumour response in mesothelioma. J Exp Clin Cancer Res. (2025) 44:51. doi: 10.1186/s13046-025-03314-w 39939955 PMC11816573

[175] Gray SG Meirson T Mutti L . Based on the real-world results from Australia, immunotherapy is not a good option for patients with mesothelioma. J Thorac Oncol. (2024) 19:541–6. doi: 10.1016/j.jtho.2024.01.016 38582546

[176] Meirson T Pentimalli F Cerza F Baglio G Gray SG Correale P . Comparison of 3 randomized clinical trials of frontline therapies for Malignant pleural mesothelioma. JAMA Netw Open. (2022) 5:e221490. doi: 10.1001/jamanetworkopen.2022.1490 35262715 PMC8908075

[177] Popat S Curioni-Fontecedro A Dafni U Shah R O’Brien M Pope A . A multicentre randomised phase III trial comparing pembrolizumab versus single-agent chemotherapy for advanced pre-treated Malignant pleural mesothelioma: the European Thoracic Oncology Platform (ETOP 9-15) PROMISE-meso trial. Ann Oncol. (2020) 31:1734–45. doi: 10.1016/j.annonc.2020.09.009 32976938

[178] Fennell DA Ewings S Ottensmeier C Califano R Hanna GG Hill K . Nivolumab versus placebo in patients with relapsed Malignant mesothelioma (CONFIRM): a multicentre, double-blind, randomised, phase 3 trial. Lancet Oncol. (2021) 22:1530–40. doi: 10.1016/S1470-2045(21)00471-X 34656227 PMC8560642

[179] Correale P Pentimalli F Nardone V Giordano A Mutti L . CONFIRM trial: what is the real efficacy of second-line immunotherapy in mesothelioma? Lancet Oncol. (2022) 23:e13. doi: 10.1016/S1470-2045(21)00702-6 34973223

[180] Kao S-H Yan TD Lee K Burn J Henderson DW Klebe S . Accuracy of diagnostic biopsy for the histological subtype of Malignant pleural mesothelioma. J Thorac Oncol. (2011) 6:602–5. doi: 10.1097/JTO.0b013e31820ce2c7 21266919

[181] Chirieac LR Hung YP Foo WC Hofer MD VanderLaan PA Richards WG . Diagnostic value of biopsy sampling in predicting histology in patients with diffuse Malignant pleural mesothelioma. Cancer. (2019) 125:4164–71. doi: 10.1002/cncr.32416 31390057 PMC7341068

[182] Incorvaia L Fanale D Badalamenti G Barraco N Bono M Corsini LR . Programmed death ligand 1 (PD-L1) as a predictive biomarker for pembrolizumab therapy in patients with advanced non-small-cell lung cancer (NSCLC). Adv Ther. (2019) 36:2600–17. doi: 10.1007/s12325-019-01057-7 31432460 PMC6822831

[183] Tagliamento M Bironzo P Curcio H De Luca E Pignataro D Rapetti SG . A systematic review and meta-analysis of trials assessing PD-1/PD-L1 immune checkpoint inhibitors activity in pre-treated advanced stage Malignant mesothelioma. Crit Rev Oncol Hematol. (2022) 172:103639. doi: 10.1016/j.critrevonc.2022.103639 35192932

[184] Yap TA Nakagawa K Fujimoto N Kuribayashi K Guren TK Calabrò L . Efficacy and safety of pembrolizumab in patients with advanced mesothelioma in the open-label, single-arm, phase 2 KEYNOTE-158 study. Lancet Respir Med. (2021) 9:613–21. doi: 10.1016/S2213-2600(20)30515-4 33836153

[185] Quispel-Janssen J van der Noort V de Vries JF Zimmerman M Lalezari F Thunnissen E . Programmed death 1 blockade with nivolumab in patients with recurrent Malignant pleural mesothelioma. J Thorac Oncol. (2018) 13:1569–76. doi: 10.1016/j.jtho.2018.05.038 29908324

[186] Roof L Koehler K Verschraegen C . Clinical importance of molecular biomarkers in pleural mesothelioma. Cancers (Basel). (2026) 18:679. doi: 10.3390/cancers18040679 41749932 PMC12939869

[187] Sha D Jin Z Budczies J Kluck K Stenzinger A Sinicrope FA . Tumor mutational burden as a predictive biomarker in solid tumors. Cancer Discov. (2020) 10:1808–25. doi: 10.1158/2159-8290.CD-20-0522 33139244 PMC7710563

[188] Perrino M De Vincenzo F Cordua N Borea F Aliprandi M Santoro A . Immunotherapy with immune checkpoint inhibitors and predictive biomarkers in Malignant mesothelioma: Work still in progress. Front Immunol. (2023) 14:1121557. doi: 10.3389/fimmu.2023.1121557 36776840 PMC9911663

[189] Zhang M Luo J-L Sun Q Harber J Dawson AG Nakas A . Clonal architecture in mesothelioma is prognostic and shapes the tumour microenvironment. Nat Commun. (2021) 12:1751. doi: 10.1038/s41467-021-21798-w 33741915 PMC7979861

[190] Lievense LA Bezemer K Cornelissen R Kaijen-Lambers MEH Hegmans JPJJ Aerts JGJV . Precision immunotherapy; dynamics in the cellular profile of pleural effusions in Malignant mesothelioma patients. Lung Cancer. (2017) 107:36–40. doi: 10.1016/j.lungcan.2016.04.015 27168021

[191] Lievense LA Cornelissen R Bezemer K Kaijen-Lambers MEH Hegmans JPJJ Aerts JGJV . Pleural effusion of patients with Malignant mesothelioma induces macrophage-mediated T cell suppression. J Thorac Oncol. (2016) 11:1755–64. doi: 10.1016/j.jtho.2016.06.021 27418106

[192] Principe N Stevens KLP Phung A-L McCoy M Kidman J Ismail A . Comparative analysis of Malignant pleural effusion and peripheral blood reveals unique T cell signatures associated with survival in mesothelioma patients. Oxf Open Immunol. (2026) 7:iqaf008. doi: 10.1093/oxfimm/iqaf008 41574275 PMC12823008

[193] Hou Y Zhang T Zhu K Jiang J Wang H . Early prediction of immunotherapy efficacy for advanced NSCLC based on clinical and pre-treatment contrast-enhanced CT radiomics features. Front Oncol. (2025) 15:1711402. doi: 10.3389/fonc.2025.1711402 41487592 PMC12757218

[194] Lin X Liu Z Zhou K Li Y Huang G Zhang H . Intratumoral and peritumoral PET/CT-based radiomics for non-invasively and dynamically predicting immunotherapy response in NSCLC. Br J Cancer. (2025) 132:558–68. doi: 10.1038/s41416-025-02948-z 39930148 PMC11920075

